# GATD3A, a mitochondrial deglycase with evolutionary origins from gammaproteobacteria, restricts the formation of advanced glycation end products

**DOI:** 10.1186/s12915-022-01267-6

**Published:** 2022-03-21

**Authors:** Andrew J. Smith, Jayshree Advani, Daniel C. Brock, Jacob Nellissery, Jessica Gumerson, Lijin Dong, L. Aravind, Breandán Kennedy, Anand Swaroop

**Affiliations:** 1 Neurobiology, Neurodegeneration and Repair Laboratory, National Eye Institute, National Institutes of Health, MSC0610, 6 Center Drive, Bethesda, MD 20892 USA; 2 UCD School of Biomolecular and Biomedical Science, Conway Institute, University College Dublin, Belfield, D4, Dublin, Ireland; 3 Genome Engineering Core, National Eye Institute, National Institutes of Health, 6 Center Drive, Bethesda, MD 20892 USA; 4 National Center for Biotechnology Information, National Library of Medicine, National Institutes of Health, Bethesda, MD 20894 USA

**Keywords:** Aging, Deglycase, DJ-1, PARK7, Glutamine amidotransferase, Mitochondria, Advanced glycation end product, Molecular evolution

## Abstract

**Background:**

Functional complexity of the eukaryotic mitochondrial proteome is augmented by independent gene acquisition from bacteria since its endosymbiotic origins. Mammalian homologs of many ancestral mitochondrial proteins have uncharacterized catalytic activities. Recent forward genetic approaches attributed functions to proteins in established metabolic pathways, thereby limiting the possibility of identifying novel biology relevant to human disease. We undertook a bottom-up biochemistry approach to discern evolutionarily conserved mitochondrial proteins with catalytic potential.

**Results:**

Here, we identify a Parkinson-associated DJ-1/PARK7-like protein—glutamine amidotransferase-like class 1 domain-containing 3A (GATD3A), with bacterial evolutionary affinities although not from alphaproteobacteria. We demonstrate that GATD3A localizes to the mitochondrial matrix and functions as a deglycase. Through its amidolysis domain, GATD3A removes non-enzymatic chemical modifications produced during the Maillard reaction between dicarbonyls and amines of nucleotides and amino acids. GATD3A interacts with factors involved in mitochondrial mRNA processing and translation, suggestive of a role in maintaining integrity of important biomolecules through its deglycase activity. The loss of GATD3A in mice is associated with accumulation of advanced glycation end products (AGEs) and altered mitochondrial dynamics.

**Conclusions:**

An evolutionary perspective helped us prioritize a previously uncharacterized but predicted mitochondrial protein GATD3A, which mediates the removal of early glycation intermediates. GATD3A restricts the formation of AGEs in mitochondria and is a relevant target for diseases where AGE deposition is a pathological hallmark.

**Supplementary Information:**

The online version contains supplementary material available at 10.1186/s12915-022-01267-6.

## Background

Having arisen over two billion years ago from the engulfment of an alphaproteobacterium by an eukaryotic precursor [1], mitochondria evolved beyond their fundamental role in producing ATP during oxidative phosphorylation into dynamic hubs regulating a multitude of cellular activities [2, 3]. The eukaryotic mitochondrial proteome consists of over 1000 proteins, which function in a plethora of biochemical processes including nucleotide and amino acid metabolism, protein synthesis, lipid, quinone and steroid biosynthesis, ion homeostasis, and fatty acid catabolism [4]. Despite innovative efforts to gain insight into ancestral proteins through proteomic and systems approaches [5], biochemical activities of many of the mammalian homologs have not been delineated, thus limiting our understanding of mitochondrial biology. Additionally, even with identification of hundreds of disease-causing mitochondrial genes, ~40% of diagnosed mitochondrial disorders are of unknown genetic cause, underpinning the necessity to use alternative approaches to add mechanistic depth to the uncharacterized mitochondrial proteome [6].

Given the eukaryotic mitochondrion’s proteobacterial provenance, we endeavored to take a bottom-up biochemistry approach to identify evolutionarily well-conserved predicted mitochondrial proteins with catalytic potential. Here, we report unique properties of the glutamine amidotransferase-like class 1 domain-containing (GATD) family member, GATD3A, and identify enzymatic activities relevant to human health and disease. GATD proteins classically generate ammonia by amidolysis from the glutamine amide nitrogen, transferring it to an acceptor substrate in a variety of anabolic reactions involving purine and pyrimidine synthesis [7]. Notably, GATD family members are linked to several human diseases, and in recent years, alternative functions have been reported for the GATD family member Parkinson-associated DJ-1/PARK7 (DJ-1), to which GATD3A has significant homology. The DJ-1 protein was demonstrated to possess a glutathione-independent glyoxylase III activity responsible for removing non-enzymatic chemical modifications (NECM) formed on the amine group of nucleotides and amino acids by 1,2-dicarbonyls during the Maillard reaction [8, 9]. The reactive dicarbonyls glyoxal (GO) and methylglyoxal (MGO) are byproducts of anaerobic glycolysis and lipid peroxidation and react with amines of amino acids, nucleotides, and lipids, forming early glycation intermediates. Subsequent dehydration and crosslinking of these can lead to advanced glycation end products (AGEs) [10, 11]. AGE formation is demonstrated to cause irreversible damage to many proteins, disrupting their structural and functional integrity [12].

In many conditions associated with glycation, such as hyperglycemia, aging, cancer, and neurodegeneration, there is a noted disruption of mitochondrial homeostasis and increased production of the reactive precursors GO and MGO, which in turn results in elevated molecular glycation damage as evidenced by a rise in AGE levels. Despite a correlation between glycation, malfunctioning mitochondria, and disease, the relative importance of mitochondrial glycation, as opposed to extracellular or cytosolic glycation, remains unclear [13].

Based on its predicted mitochondrial localization, homology to the Parkinson-associated DJ-1 and affinities to proteins from bacterial lineages distinct from alphaproteobacteria, we chose to focus on GATD3A (glutamine amidotransferase-like class 1 domain-containing 3A). Here, we show that GATD3A is a nucleotide and amino acid deglycase in the mitochondrial matrix where it interacts with mitochondrial protein translation machinery. Absence of GATD3A causes enhanced glycation of both ribosomal RNA and protein species and altered GATD3A expression levels influence mitochondrial dynamics. Using an evolutionary approach, our study supports a role for GATD3A as a glycation defense mechanism in mitochondria and corroborates the concept that mitochondrial glycation is an important non-enzymatic chemical modification relevant to human biology and disease.

## Results

### Mammalian GATD3A has bacterial provenance distinct from the mitochondrial progenitor

**Fig. 1:**
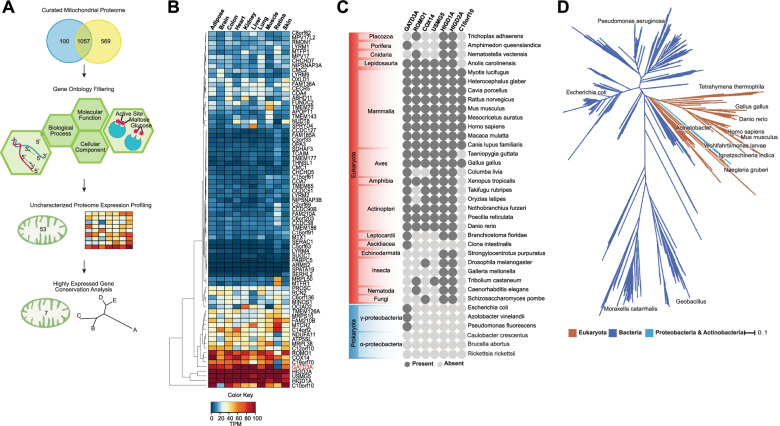
Mammalian GATD3A exhibits bacterial provenance distinct from the mitochondrial progenitor. **A** Workflow examining proteins from MitoCarta 2.0 and MitoMiner 4.0, based on biological process or molecular function. Of the 53 poorly characterized mitochondrial proteins, GATD3A was selected for further analysis based on expression profile and evolutionary conservation. **B** Expression analysis of genes encoding uncharacterized mitochondrial proteins across human tissues in GTEx and retinal RNAseq data. We selected the cluster of highly expressed genes for conservation analysis. TPM denotes transcripts per million. **C** Evolutionary conservation of seven uncharacterized predicted mitochondrial proteins—GATD3A, ROMO1, COX14, USMG5, HIGD1A, HIGD2A, and C10orf10, exhibiting high expression of corresponding genes in human tissues. None of the proteins barring HIGD1A and HIGD2A have a close alphaproteobacterial homolog. GATD3A is the only protein with homologs in gammaproteobacteria and some other bacterial lineage. **D** Phylogenetic tree showing the evolutionary relationships of GATD3A homologs in eukaryotes as well as bacteria. The clade in blue color represents bacteria, clade in brown color represents eukaryota, and clade in light blue color represents the following bacteria *Paludibacterium purpuratum*, *Chitinimonas sp. R3-44*, *Crenobacter luteus*, *Vogesella sp. YM-1*, *Microvirgula*, and *Gulbenkiania mobilis*. Branch lengths (represents the evolutionary time between two nodes) are represented by scale bar (0.1)

We initially hypothesized that identification of evolutionarily conserved but as yet uncharacterized mitochondrial proteins would help us prioritize in-depth molecular characterization. To select a group of proteins for conservation analysis, we first compared two well-curated human mitochondrial proteome compendia [14, 15] and carried out gene ontology analysis of the common proteins. Of the 1057 proteins present in both, 53 proteins had no known biological process or molecular function, representing ~5% of the human mitochondrial proteome (Fig. 1A). We then examined their expression across human tissues using RNAseq data to select for genes with ubiquitous expression [16, 17] (Fig. 1B). We chose a cluster of 7 highly expressed genes (*ROMO1, GATD3A, COX14, USMG5, HIGD1A, HIGD2A, C10orf10*) for conservation analysis and used BLAST (Basic Local Alignment Search Tool) with their respective predicted protein sequences to identify which genes have homologs in model organisms. We found GATD3A to be widely conserved in both eukaryotes and bacteria when compared to the other 6 genes which were restricted to eukaryotes or showed only a limited presence in bacteria (HIGD1A and HIGD2A have widespread alphaproteobacterial homologs; Fig. 1C). Notably, our analysis indicated that unlike HIGD1A and HIGD2A, GATD3A is present in a large group of bacteria but is absent in alphaproteobacteria. To further explore evolutionary conservation of GATD3A in prokaryotes, we carried out phylogenetic analysis for homologous sequences of GATD3A in different organisms using Refseq protein database. We observed eukaryotic GATD3A orthologs nested within a vast radiation of bacterial GATD proteins with closest homologs in gammaproteobacteria (e.g., *Ignatzschineria*), betaproteobacteria (e.g., *Microvirgula*), and actinobacteria (e.g., *Chitinimonas sp*. R3-44) (Fig. 1D). Given this unique evolutionary conservation profile compared to other ubiquitously expressed uncharacterized mitochondrial factors, we surmised GATD3A to be an attractive predicted mitochondrial protein for functional characterization.

### GATD3A localizes to the mitochondrial matrix

**Fig. 2:**
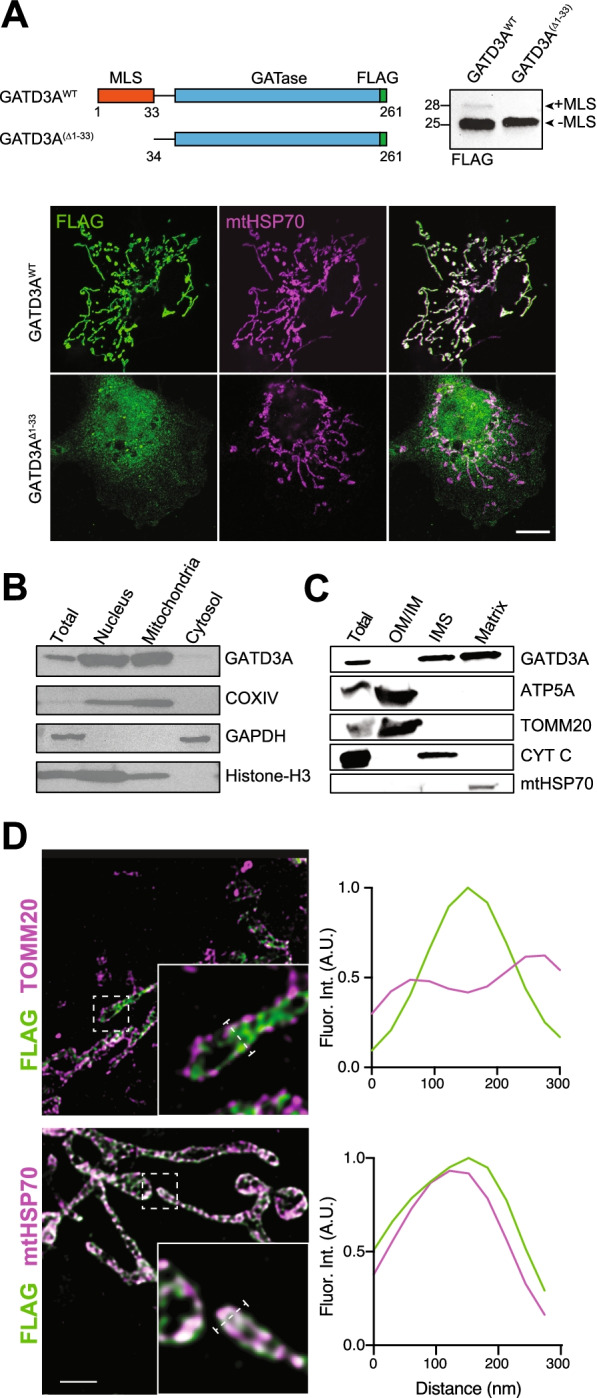
GATD3A is localized in the mitochondrial matrix. **A** A schematic of GATD3A^WT^ and GATD3A^Δ1-33^ expression constructs with FLAG-tag. Immunoblot analysis revealed two bands for GATD3A^WT^, the larger being GATD3A with its mitochondrial localization signal (MLS) uncleaved. GATase (glutamine amidotransferase), (scale = 10 μm). **B** Subcellular fractionation reveals absence of GATD3A from a post-mitochondrial cytosolic fraction. **C** GATD3A in heart mitochondrial membrane, intermembrane and matrix fractions. OM/IM, outer/inner membrane; IMS, intermembrane space. **D** Fluorescence intensity profile across a mitochondrion using stimulated emission depletion (STED) super-resolution microscopy. GATD3A (green) colocalizes specifically with the mitochondrial matrix protein mtHSP70 (magenta), and not the outer mitochondrial membrane import protein TOMM20 (magenta) (scale = 1 μm)

Given the paucity of molecular evidence for GATD3A subcellular localization [18], we first cloned the human GATD3A with and without the predicted mitochondrial localization signal (MLS) (GATD3A^Δ1-33^), carrying a FLAG-tag at the C-terminus and expressed these in COS7 cells under the control of a CMV promoter (Fig. 2A). Using immune-based co-localization methods with the mitochondrial matrix protein mt-Hsp70, we confirmed the mature GATD3A^WT^ protein localizes to the mitochondrion, where the signal peptide is then cleaved. Removal of the predicted MLS causes mislocalization of GATD3A. Subcellular fractionation showed GATD3A is enriched in the mitochondria and largely absent from a post-mitochondrial cytosolic fraction (Fig. 2B). To further confirm the localization of GATD3A in tissue, we performed hypotonic fractionation of the heart mitochondria, which exhibit high GATD3A expression, by separating these into matrix, intermembrane, and membrane fractions. GATD3A was largely concentrated in the mitochondrial matrix with a small proportion in the intermembrane space (Fig. 2C). We then used super-resolution stimulated emission depletion microscopy (STED) of transfected COS-7 cells, confirming that GATD3A^WT^ protein is localized to the mitochondrial matrix and colocalized with mtHsp70. GATD3A was undetected in the mitochondrial outer membrane based on the distinct fluorescence intensity profile with the outer membrane marker TOMM20 (Fig. 2D). Thus, GATD3A is an evolutionarily conserved mitochondrial matrix resident protein in mammalian tissues and cells.

### GATD3A and DJ-1/PARK7 amidolysis domains are required for deglycation of nucleotides and amino acids

**Fig. 3:**
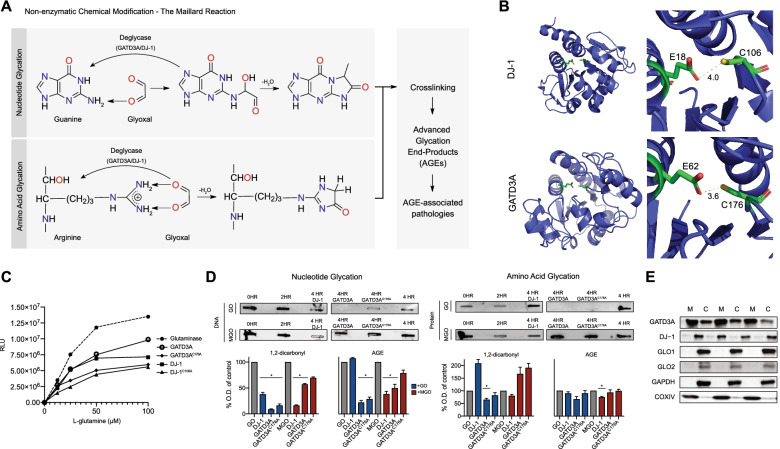
Amidolysis domains of GATD3A and DJ-1 are required for deglycation of nucleotides and amino acids. **A** Genesis of advanced glycation end products (AGE). The Maillard reaction involving glyoxal results in non-enzymatic glycation of nucleotides (e.g., guanine) and amino acids (e.g., arginine), which is reversed by GATD3A/DJ-1 deglycation. AGE are formed by subsequent dehydration and crosslinking of nucleotides and amino acids with Maillard adducts, leading to associated pathologies. **B** Glutamine amidotransferase active sites in GATD3A and DJ-1, showing high structural homology and conservation of key predicted catalytic residues, E18 and C106 in DJ-1, and E62 and C176 in GATD3A. **C** Luciferase-linked glutamate detection assay demonstrating the capacity of GATD3A and DJ-1 to hydrolyze glutamine. Glutaminase is used as a positive control. Amidolytic activity is reduced with mutation of conserved catalytic cysteine residues C176 and C106 in GATD3A and DJ-1, respectively. Representative results of *n* = 3 assays are shown. **D** Immunoreactivity assay of glyoxal (blue) or methylglyoxal (MGO) (red) glycated plasmid DNA or γ-globulin protein. Following 2 h of incubation with either MGO or GO, samples were treated with recombinant DJ-1 (4HR DJ-1), GATD3A (4HR GATD3A), GATD3A C176A (4HR GATD3A C176A), or a no enzyme control (4HR) for a further 2 h. Optical density (O.D.) of immunodetected 1,2-dicarbonyl and AGE (images in Additional file 1, Fig. S1D) is calculated for three replicate experiments and normalized to the no enzyme control (4HR) (*p* < 0.01, two-tailed Student’s *t* test, *n* = 3, error bars = SEM). The 0HR timepoint was omitted from analysis due to high variability in glycation levels. Glyoxal—GO, Methylglyoxal—MGO. Full blots are shown in SI2. **E** Detection of glutathione-dependent glyoxylases GLO-1 and GLO-2 and deglycases DJ-1 and GATD3A in mouse heart mitochondria (M) and post-mitochondrial cytosol (C) (*N* = 3 mice). COXIV is used as a mitochondrial control, GAPDH is used as a cytosolic control. Extended analysis is included in Additional file 1, Fig. S4A

Structural analysis of GATD3A revealed a glutamine amidotransferase (GATase) domain, common among all proteins belonging to the GATD family. GATD proteins catalyze the amidolysis of glutamine amide nitrogen and transfer it to an acceptor substrate in anabolic reactions including purine, pyrimidine, amino acid, and co-enzyme synthesis [7, 19, 20]. Significant sequence homology between both prokaryotic and eukaryotic homologs suggested that GATD3A possesses catalytic activity similar to another GATD family member, Parkinson-associated protein DJ-1/PARK7, which removes NECMs formed during the Maillard reaction [21, 22]. Importantly, this activity is suggested to occur via an amidolytic process. The 1,2-dicarbonyls, GO and MGO, are highly reactive byproducts of anaerobic glycolysis and lipid peroxidation. Specifically, the reactive 1,2-dicarbonyl glyoxal is formed endogenously through retroaldol condensation of glucose, the auto-oxidation of glycoaldehyde, and lipid peroxidation of polyunsaturated fatty acids through hydroperoxide formation. The non-enzymatic glycation of free amino groups by aldehydes, termed the Maillard reaction, forms glycation intermediates. Successive rearrangements of intermediate compounds and crosslinking of these adducts then generates advanced glycation end products (AGEs) (Fig. 3A), which compromise the functionality of respective macromolecules, leading to AGE-related pathologies [11, 23]. Structural analysis of conserved amidotransferase catalytic residues in nucleophilic elbows, specifically E18 and C106 in DJ-1 and E62 and C176 in GATD3A, supported the existence of amidolytic active sites (Fig. 3B). We hypothesized that DJ-1 and GATD3A behave as classical GATases and can hydrolyze glutamine. Using recombinant protein (Additional file 1, Fig. S1A) in an in vitro end-point luciferase-based glutamate detection assay, we demonstrate that GATD3A and DJ-1 can hydrolyze free glutamine and that mutation of the conserved catalytic cysteine residues C176 in GATD3A and C106 in DJ-1 results in diminished amidolytic activity (Fig. 3C).

The DJ-1 deglycase activity is hypothesized to be dependent on an amidolytic reaction [21]. We next hypothesized that GATD3A can reverse the formation of early glycation intermediates, similar to DJ-1. We used 1,2-dicarbonyl immunodetection methods to identify the glycation status of plasmid DNA and γ-globulin protein species following exposure to the reactive dicarbonyls GO and MGO, which were subsequently incubated with either recombinant GATD3A or DJ-1 (Additional file 1, Fig. S1B). Incubation of DNA with the reactive 1,2-dicarbonyl glyoxal leads to a time-dependent increase in DNA glycation levels, as evidenced by 1,2-dicarbonyl immunodetection (Additional file 1, Fig. S1C). When comparing to a no-enzyme glycated control, our results show that GATD3A deglycates both DNA and protein species chemically modified primarily with GO, whereas DJ-1 has a greater specificity for samples modified by MGO (Fig. 3D). GATD3A and DJ-1 reduce the generation of AGE on DNA and protein species compared to no enzyme controls at 4 h (Fig 3D; Additional file 1, Fig. S1C, D). GATD3A^C176A^ mutant exhibits lower activity compared to the wildtype GATD3A, demonstrating that this conserved cysteine residue is involved in modulating deglycase activity.

Mammalian cells have multiple defense mechanisms for the removal of potentially damaging aldehydes including glutathione-dependent glyoxylases, and glutathione-independent deglycases. We next wanted to examine if any of these defense mechanisms exist within mitochondria under physiological conditions. Cellular fractionation studies of heart tissue, which has high GATD3A expression, reveal that GATD3A is the only glycation defense mechanism within mitochondria (Fig. 3E). We thus conclude that GATD3A and DJ-1 are nucleotide and amino acid deglycases with greater affinity for chemical modifications caused by GO and MGO, respectively. This deglycation of early Maillard adducts is achieved through their amidolytic activity, which is modulated by highly conserved cysteine residues. Notably, GATD3A appears to be the only primarily mitochondrial deglycase.

### GATD3A interacts with mitochondrial RNA and translation machinery and is necessary for preventing AGE formation

**Fig. 4:**
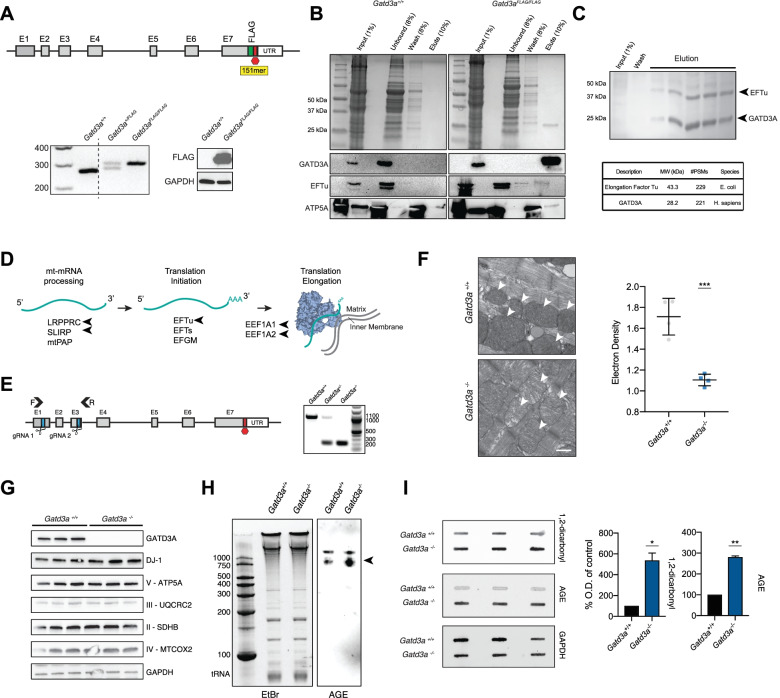
Interaction with mitochondrial mRNA and translation machinery shows GATD3A is necessary for preventing AGE formation. **A** CRISPR-Cas9-mediated insertion of a FLAG allele in the C-terminus of GATD3A using a donor 151mer oligonucleotide. PCR genotyping shows insertion of 24-bp fragment in the knock-in mice. Immunoblot analysis detects FLAG epitope in knock-in mice only. GAPDH is used as a loading control. **B** Co-elution of EFTu and ATP5A with GATD3A by FLAG immunoprecipitation from *Gatd3a*^FLAG/FLAG^ mouse heart mitochondria. **C** Coomassie gel showing consistent co-elution of the bacterial elongation factor EFTu with recombinant human GATD3A:6X-His from *E. coli* by fast protein liquid chromatography. **D** Model showing GATD3A interactors (arrowheads) at the interface of mitochondrial mRNA maturation and stabilization, and mitochondrial protein translation (representative of *n* = 3 mice used in immunoprecipitation). **E** CRISPR-mediated removal of a part of *Gatd3a* coding region between exons 1 and 3, leading to total loss of the mature GATD3A protein. PCR genotyping shows a large deletion of ~850 bp in the knockout allele. gRNA—guideRNA, E—exon, F—forward primer, R—reverse primer, UTR—untranslated region. **F** Transmission electron microscopy (TEM) indicates significant loss of electron dense mitochondrial structure (arrowheads) compared to electron density of muscle fibers in left ventricle cardiac tissue of 1-year-old *Gatd3a*^−*/*−^ mice (*p* = ***<0.001, two-tailed Student’s *t* test, *n* = 3 per genotype, *N* = 4 analyzed sections per genotype, scale bars = ±SD, TEM scale bar = 500 nm). **G** Aged (1 year old) GATD3A knockout mice do not have differential protein expression of deglycase DJ-1, or oxidative phosphorylation components. GAPDH is used as a loading control. **H** Augmented AGE immunoreactivity of mitochondrial ribosomal 16S and 12S RNA species in one-year-old *Gatd3a*^−*/*−^ mouse heart ventricle. The arrowhead denotes the increased AGE reactivity of the 12S RNA in *Gatd3a*^−*/*−^ mouse. Representative blot of *n* = 3 mice per genotype. **I** Enhanced 1,2-dicarbonyl and AGE immunoreactivity in heart tissue of one-year-old *Gatd3a*^−*/*−^ mice as detected by slot blot assay. GAPDH is used as a loading control. (*n* = 3 per genotype, *p* = *<0.05, **<0.01, two-tailed Student’s *t* test, +SEM)

To decipher a biological role in mitochondria, we examined GATD3A interacting proteins using a custom generated *Gatd3a*^FLAG/FLAG^ mouse. Insertion of a FLAG epitope tag (DYKDDDDK) to the C-terminus of GATD3A was achieved through CRISPR-mediated homologous recombination (HR) with a 151-nucleotide-long single-strand DNA as the donor template (Fig. 4A) [24]. FLAG co-immunoprecipitation studies using heart tissue identified several proteins associated closely with the mitochondrial inner membrane that function in ATP synthesis and transport (ATP5A, ATP5B, ADP/ATP Translocase 1/2), and mitochondrial mRNA (mt-mRNA) processing, protein translation, and elongation (LRPPRC, SLIRP, EFTu, EEF1A1, EEF1A2) (Fig. 4B; Additional file 1, Fig. S2; Table 1). We chose to focus on a function for a deglycase at the interface of mt-mRNA processing and translation since the interaction of GATD3A with mitochondrial elongation factor EFTu appears to be conserved from prokaryotes. This was evidenced by consistent co-elution of the bacterial EFTu with the recombinant human GATD3A when expressed in *Escherichia coli* (Fig. 4C). EFTu in mammalian mitochondria descends from its cognate in the alphaproteobacterial mitochondrial progenitor and is responsible for the GTP-dependent binding of aminoacyl-tRNAs to the A site of ribosomes during protein synthesis (Fig. 4D) [25].

**Table 1:** The proteins identified by LC-MS/MS following GATD3A:FLAG immunoprecipitation

Accession	Description	# Peptides	# PSMs
Gatd3a	Glutamine amidotransferase-like class 1 domain-containing protein 3A	24	351
Lrpprc	Leucine-rich PPR motif-containing protein, mitochondrial	33	79
Hspa9	Stress-70 protein, mitochondrial	25	77
Ighg1	Ig gamma-1 chain C region, membrane-bound form	15	71
Atp5b	ATP synthase subunit beta, mitochondrial	19	56
Hspa5	Endoplasmic reticulum chaperone BiP	21	51
Aldh1l2	Mitochondrial 10-formyltetrahydrofolate dehydrogenase	20	48
Tufm	Elongation factor Tu, mitochondrial	14	36
Igkc	Immunoglobulin kappa constant 2	4	32
Cycs	Cytochrome c, somatic	10	31
Hbb-b1	Hemoglobin subunit beta-1	7	29
Hba	Hemoglobin subunit alpha	4	29
Atp5a1	ATP synthase subunit alpha, mitochondrial	11	26
Mdh2	Malate dehydrogenase, mitochondrial	10	25
Flna	Filamin-A	12	25
Acadl	Long-chain-specific acyl-CoA dehydrogenase, mitochondrial	9	23
Prmt5	Protein arginine N-methyltransferase 5	9	21
Hadha	Trifunctional enzyme subunit alpha, mitochondrial	8	19
Aco2	Aconitate hydratase, mitochondrial	7	17
Wdr77	Methylosome protein 50	7	17
Ppib	Peptidyl-prolyl cis-trans isomerase B	5	16
Hspd1	60 kDa heat shock protein, mitochondrial	7	16
Hadhb	Trifunctional enzyme subunit beta, mitochondrial	7	16
Slc25a4	ADP/ATP translocase 1	8	15
Vcp	Transitional endoplasmic reticulum ATPase	8	14
Hbb-b2	Hemoglobin subunit beta-2	5	13
Hspa8	Heat shock cognate 71 kDa protein	5	12
Fkbp3	Peptidyl-prolyl cis-trans isomerase FKBP3	6	11
Pcca	Propionyl-CoA carboxylase alpha chain, mitochondrial	5	11
Dsp	Desmoplakin	10	11
Eef1a2	Elongation factor 1-alpha 2	5	10

Only proteins with peptide spectral match (PSM) of > 10 are shown. Co-immunoprecipitation was performed three times using anti-FLAG antibody from heart mitochondria of *Gatd3a*^FLAG/FLAG^ mice

Given the association of glycation with aging, we investigated the glycation status and health of the aged heart in the absence of GATD3A. A *Gatd3a*^−/−^ mouse was generated using CRISPR with two gRNAs flanking the genomic region of exon 1 to exon 3. A deletion allele, lacking 865 base pairs in one of the F0 founders, resulted in the loss of essential coding and splice sites, generating a truncated transcript containing a premature stop codon (Fig. 4E). In the aged *Gatd3a*^−/−^ heart, we detected a significant decrease in mitochondrial electron density by transmission electron microscopy (TEM) without concomitant changes in protein levels (Fig. 4F, G), highlighting a necessity for a resident mitochondrial deglycase.

Based on the preceding results, we hypothesized that GATD3A is responsible for the reversal of NECMs at the interface of mitochondrial RNA processing and protein translation. We then evaluated RNA and proteins in the aged *Gatd3a*^−/−^ mouse heart and identified enhanced accumulation of AGEs on both mitochondrial 16S and 12S ribosomal RNA species and proteins (Fig. 4H, I). We postulated that additional carbonyl defense mechanisms (e.g., GLO1, GLO2, or DJ-1) could compensate for the loss of GATD3A, either by enhanced expression or via translocation to mitochondria. Examination of GATD3A, GLO-1, GLO-2, and DJ-1 expression levels in mitochondrial and cytosolic fractions of young (2 months) and aged (23 months) *Gatd3a*^−/−^ and wildtype littermates showed no significant changes in response to loss of GATD3A (Additional file 1, Fig. S3). Notably, we detected a small but significant increase in cytosolic GLO-1 levels in aged mice independent of the absence of GATD3A. The observed correlative increase in protein and nucleotide glycation status with a reduction in mitochondrial electron density suggest a role for GATD3A in removing damaging non-enzymatic chemical modifications in vivo to support mitochondrial homeostasis.

### Altered GATD3A expression influences mitochondrial dynamics

**Fig. 5:**
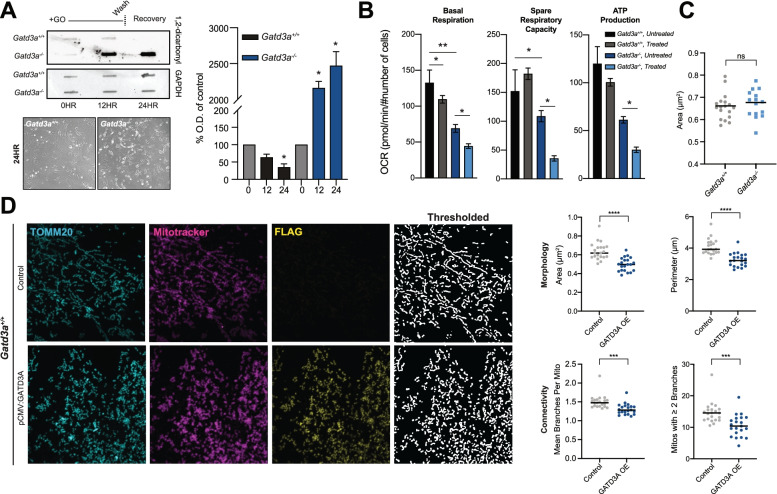
GATD3A influences mitochondrial dynamics. **A** 1,2-dicarbonyl slot blot immunoreactivity pulse-chase assay and cellular morphology of glyoxal (GO)-treated *Gatd3a*^+/+^ or *Gatd3a*^−/−^ MEFs. GAPDH is used as a loading control (*p* = *<0.05, two-tailed Student’s *t* test, *n* = 4, +SEM). **B** Oxygen consumption rate is decreased in *Gatd3a*^−*/*−^ mouse embryonic fibroblasts (MEFs) compared to wildtype. Treatment with methylglyoxal (0.2 mM) for 24 h prior to assay exacerbates observed phenotype in knockout cells. Data is normalized to cell number (*p* = *<0.05, *p* = *<0.01, two-tailed Student’s *t* test, *n* = 3 biological replicates, *N* = 4 technical replicates per plate, *N* = 3 replicates per genotype, error bars ±SEM). **C** Mitochondrial morphology is not significantly disrupted in *Gatd3a*^−*/*−^ MEFs (*N* = 21 per genotype, two-tailed Welch’s *t*-test, lines represent mean value, extended data in Additional file 1, Fig. S4B). **D** Overexpression of GATD3A in *Gatd3a*^*+/+*^ MEFs leads to reduced mitochondrial content and fragmented mitochondrial network (*N* = 21 per condition, *p* = ****<0.0001, ***<0.001, two-tailed Welch’s *t*-test, lines represent mean value)

To further elucidate the function of GATD3A in mitochondrial biology, we generated mouse embryonic fibroblasts (MEFs) from the *Gatd3a*^−/−^ mouse and sought to validate GATD3A deglycase activity in this closed system by performing a pulse-chase experiment. *Gatd3a*^−/−^ MEFs treated with exogenous GO exhibited enhanced protein glycation levels compared to the wildtype cells following both treatment and recovery (Fig. 5A), confirming GATD3A’s role as a deglycase. We then examined whether the loss of GATD3A disrupted mitochondrial oxygen consumption in MEFs. We observed a significant decrease in basal respiration, spare capacity, and ATP production associated with the loss of GATD3A. Additionally, we detected a further reduction of all mitochondrial respiratory parameters in *Gatd3a*^−/−^ MEFs following treatment with methylglyoxal (Fig. 5B). Given the reduction in electron density observed in the aged knockout mouse heart, we postulated that *Gatd3a*^−*/*−^ MEFs may display a quantifiable change in mitochondrial morphology but did not observe significant differences in the area measured with immunofluorescence methods (Fig. 5C; Additional file 1, Fig. S4A).

Curiously, overexpression of GATD3A in wildtype MEFs resulted in altered mitochondrial dynamics. We observed extensive fragmentation and what appeared as potential increased mitochondrial mass relative to untransfected and knockout cells (Fig. 5D, Additional file 1, Fig. S4B). We hypothesized this to be caused by an induction of mitochondrial biogenesis, as indicated by the formation of “mega-mitochondria” upon overexpression of a zebrafish *GATD3A* paralog [26]. To assess the induction of mitochondrial biogenesis, we quantified the relative mitochondrial DNA (mtDNA) copy number compared to nuclear DNA but no significant differences were detected between wildtype and knockout, or wildtype overexpressing GATD3A (Additional file 1, Fig. S4C). We did however observe significantly increased fragmentation and reduced mitochondrial area relative to wildtype cells following GATD3A overexpression (Fig. 5D). This fragmentation was phenocopied in human HEK293 cells, which did not display induction of mitochondrial biogenesis assessed via mtDNA copy number, similar to MEFs (Additional file 1, Fig. S4D, E). We examined expression of known mitochondrial biogenesis factors and identified a modest increase in *PGC1α* expression upon GATD3A overexpression (Additional file 1, Fig. S4F) and a concurrent though modest induction of nuclear and mitochondria-encoded factors (NDUFB8, MTCO2) (Additional file 1, Fig. S4G). Our data do not support previous findings indicating a role for GATD3A in mitochondrial biogenesis, yet a role of GATD3A can be deduced in mitochondrial dynamics. Given our observations in aged mice, these results suggest a potential role of GATD3A under stress conditions, which support the accumulation of early glycation intermediates.

## Discussion

Advances in cataloging the mitochondrial proteome coupled with revolutions in “omics”-based technologies and DNA editing have helped functionalize the uncharacterized mitochondrial proteome. Recent efforts primarily apply forward genetic approaches to attribute functions to proteins in established pathways, leaving a missed opportunity to discover novel mitochondrial biology [27]. A surprisingly large number of mitochondrial proteins still lack any functional characterization [5]. Importantly, many mitochondrial disorders lack any molecular diagnosis [6]. A core group of mitochondrial proteins with indispensable functions are largely conserved throughout evolution in eukaryotes [28]. In addition, 20–30% of mitochondrial proteomes have specific phylogenetic relationships to proteobacterial cognate, and many of these have potential endosymbiotic origins from the mitochondrial progenitor [29, 30]. We therefore hypothesized that proteins important for mitochondrial function and potentially human health would be expressed across a variety of human tissues and be well-conserved evolutionarily. We were intrigued by a ubiquitously expressed mitochondrial factor GATD3A with closest bacterial homologs in betaproteobacteria, gammaproteobacteria, and actinobacteria, but not in alphaproteobacteria, despite querying an extensive genomic dataset. The absence of conserved gene neighborhood for the bacterial orthologs (e.g., ElbB) suggests it likely functions as a standalone protein and not as part of a coordinated isoprenoid biosynthetic pathway as previously suggested [31]. Thus, GATD3A was likely acquired from a bacterial source distinct from the alphaproteobacterial progenitor in a single event of lateral gene transfer and recruited for a mitochondrial function early in eukaryotic evolution as evidenced by its presence in ciliates (*Tetrahymena thermophila*), rhizarians (*Reticulomyxa filosa*), and more basal forms (*Naegleria gruberi*). Interestingly, there appears to be a GATD3A secondary gene loss in fungi (e.g., *Saccharomyces cerevisiae*), which are more closely related to animals; the loss is presumably compensated by another family member, such as DJ-1.

STED microscopy of GATD3A:FLAG in COS-7 cells coupled with *Gatd3a*^FLAG/FLAG^ mitochondria subfractionation confirmed the localization of GATD3A to the mitochondrial matrix, with a smaller proportion in the intermembrane space. Notably, DJ-1 is shown to localize to the mitochondrial matrix and intermembrane space under specific conditions [32, 33] though some controversy remains [34]. Unlike DJ-1, GATD3A is largely a resident mitochondrial matrix protein. Under physiological conditions, glutathione-dependent glyoxylases are sufficient for the removal of detrimental aldehydes. During oxidative stress, reactive molecules such as GO and MGO accumulate [35]. We suggest that DJ-1 and GATD3A act as an additional defense mechanism to reverse the formation of early glycation intermediates on nucleotides and amino acids. Importantly, our data shows that GATD3A is likely a resident glycation defense mechanism for reversing NECMs on important biomolecules, previously thought to be irreversible [36].

Sequence analysis of GATD3A and DJ-1 bacterial and eukaryotic homologs confirmed the conservation of cysteine and glutamic acid residues in catalytic regions, which are predicted to be glutamine amidotransferase class 1-like (GATase) domains. GATases catalyze the amidolysis of ammonia from glutamine, transferring it to a substrate to form a new carbon-nitrogen group and produce glutamate as a byproduct. Some GATases serve important functions including the synthesis of amino acids, purine and pyrimidine nucleotides, co-enzymes (nicotinamide adenine dinucleotide), amino sugars (glucosamine), and antibiotics [7, 19, 20]. Class I glutamine amidotransferases rely on a catalytic Cys-His-Glu triad [37] to perform its amidolytic function. Human GATD3A has two potential conserved catalytic His residues (His62 and His251) located proximal to the predicted active site in a nucleophilic elbow (data not shown), similar to His126 in DJ-1, which is required for its glutathione-independent glyoxylase activity [8]. Using free L-glutamine as a substrate, we show that GATD3A and DJ-1 can hydrolyze glutamine and that this activity is modulated by conserved cysteine residues, C176 and C106, in the respective proteins. Mutagenesis of C106 in DJ-1 is reported to eliminate any enzymatic activity [22]; however, we demonstrate diminished activities for both DJ-1 and GATD3A mutants which may be attributable to potential contaminants in our purification.

Structural analyses did not suggest the presence of additional ATP-dependent synthetase domains as observed in other proteins with GATase domains such as GMP synthetase or carbamoyl-phosphate synthetase [20, 38, 39], indicating a potential alternative function dependent on its amidolysis capacity. An *Escherichia coli* homolog of DJ-1, Hsp31, was first demonstrated to be a glutathione (GSH)-independent glyoxylase (glyoxylase III) capable of converting methylglyoxal (MGO) to D-Lactate [40]. More recently, DJ-1 was shown to play a more physiologically relevant role by deglycating amino groups of nucleotides and amino acids which have undergone non-enzymatic glycation by 1,2-dicarbonyls glyoxal and methylglyoxal in the Maillard reaction [21, 22, 41]. Using an immune-based 1,2-dicarbonyl detection method, we confirmed previous findings about DJ-1 acting as a deglycase and show a specificity of GATD3A to deglycate nucleotide and amino acid residues non-enzymatically glycated by glyoxal. Our finding is consistent with observations of a glyoxal-specific activity for the GATD3A bacterial homolog ElbB [42]. Furthermore, we demonstrate that the reversal of early glycation by GATD3A and DJ-1 prevents the formation of AGE, which result from dehydration and crosslinking of glycation adducts [43]. Given the diversity of stable advanced glycation end products, our findings are limited to pan-AGE products and do not differentiate between crosslinked and non-crosslinked species.

FLAG co-immunoprecipitation assays using *Gatd3a*^FLAG/FLAG^ heart mitochondria identified proteins of the mitochondrial inner membrane involved in ATP synthesis, mitochondrial mRNA processing (LRPPRC, SLIRP), and protein translation (EFTu). GATD3A is reported to exhibit RNA-binding properties [18] and knockdown of GATD3A can increase the mtDNA-encoded ND1-3 RNA levels, potentially through post-transcriptional modification [44], further highlighting a role for GATD3A in mt-mRNA regulation. The identification of a conserved interaction with EFTu homologs coupled with its ability to deglycate both amino acid and nucleotide species are suggestive of a potential role of GATD3A in mt-mRNA elongation and maturation during protein translation.

An imbalance between the production of glycation precursors (1,2-dicarbonyls) and their removal results in carbonyl stress, leading to accumulation of glycation damage [45]. Mitochondrial proteins, mtDNA, and lipids are all targets for glycation [46–48] and suggested to be more susceptible to glycation than those in other cellular compartments [47, 49]. Thus, GATD3A can be reasonably hypothesized as a resident mitochondrial deglycase. Concordantly, we observed augmented AGE immunoreactivity of mitochondrial ribosomal 16S and 12S RNA species and enhanced protein 1,2-dicarbonyl and AGE immunoreactivity in the heart left ventricle of aged *Gatd3a*^−/−^ mice, which can potentially result in breakdown of their electron dense cristae structure, a phenotype indicative of dilated cardiomyopathy [50]. Interestingly, GATD3A is significantly downregulated in patient fibroblasts with a specific form of inheritable dilated cardiomyopathy with ataxia (DCMA) caused by mutations in the gene *DNAJC19* [51]. Patients with dilated cardiomyopathy (DCM) display fragmented mitochondrial morphology, similar to the *Gatd3a*^−/−^ mouse. Additionally, a variety of mutations in EFTu result in combined oxidative phosphorylation deficiency 4 (COXPD4), with DCM being a common clinical phenotype in patients [52, 53]. Our results thus indicate a role of GATD3A in supporting mitochondrial homeostasis.

Glycation and AGE modifications are one of many mechanisms for aging-associated phenotypes and complications of hyperglycemia in diabetes [43, 54, 55]. Despite a correlation between glycation, malfunctioning mitochondria, and disease, the relative importance of mitochondrial glycation, as opposed to extracellular or cytosolic glycation, remains unclear [13]. Loss of GATD3A in vivo leads to disruption of the mitochondrial reticular network, similar to patients with DCMA, which could be restored through the reintroduction of GATD3A [51]. In addition, overexpression of GATD3A in HEK293 cells and MEFs resulted in fragmented mitochondria. However, our data did not provide clear evidence of an induction of mitochondrial biogenesis in response to increased GATD3A as has been previously reported in zebrafish [26]. Future studies should focus on deciphering the contribution of GATD3A and mitochondrial carbonyl balance to mitophagy and mitochondrial protein proteasomal degradation. We propose that mitochondrial carbonyl balance is partly modulated by GATD3A and is an important contributor for maintaining mitochondrial homeostasis.

## Conclusions

This study undertook an evolutionary approach to identify well-conserved uncharacterized mitochondrial proteins, with an aim to elucidate novel biology. We focused on GATD3A, which displayed a unique phyletic profile widely distributed across eukaryotes and bacteria, ubiquitous expression in human, and significant homology with the Parkinson disease-associated protein DJ-1/PARK7. We demonstrated GATD3A’s localization primarily within the mitochondrial matrix, which is dependent on an N-terminal signaling peptide. We showed that the predicted glutamine amidotransferase-like domains of DJ-1 and GATD3A are capable of hydrolyzing glutamine and that this amidolysis activity is used for the deglycation of early intermediate glycated amino acids and nucleotides. This study confirmed a previously disputed finding regarding DJ-1 deglycase activity and provided the first characterization of GATD3A’s catalytic activity. Profiling GATD3A protein interactors also revealed conservation of binding important factors involved in mitochondrial translation. Additionally, aged mouse cardiac tissue displayed enhanced RNA and protein glycation in the absence of GATD3A, further underpinning the correlation of GATD3A expression with mitochondrial glycation status. This work suggests an involvement of GATD3A in mitochondrial dynamics.

Our study establishes that GATD3A acts as a resident mitochondrial deglycase removing non-enzymatic chemical modifications formed between amino groups of biomolecules and dicarbonyl byproducts produced from glucose and lipid oxidation. We suggest that GATD3A acts as a defense mechanism against glyoxal-based amino modifications, complementing the glutathione-dependent glyoxylase system in preventing the formation of AGE in mitochondria. GATD3A function can therefore be targeted for treatment of diseases where glycation and mitochondrial dysfunction are hallmarks.

## Methods

### Gene expression and gene ontology analysis

The mitochondrial proteome from Mitocarta and Mitominer databases were compared to identify unique and common proteins. The proteins present in both the databases were further analyzed for gene ontology analysis from The Gene Ontology Consortium [56]. The proteins not showing any biological process or molecular function were further considered for RNAseq profiles in different human tissues from GTEx consortium and the retina transcriptome dataset [16, 17]. The proteins with high expression were further explored for evolutionary conservation analysis. BLAST (Basic Local Alignment Search Tool) was carried out to identify the presence of homologs in different organisms.

### Phylogenetic analysis and molecular modelling

BLAST was carried out for the GATD3A mouse protein sequence with RefSeq protein database to identify the presence of homologs in different organisms and fasta files of these homologous sequences were downloaded. Multiple sequence alignment was carried out for all the sequence fasta files using MAFFT tool (Version 7.407) [57]. The output file generated after multiple sequence alignment using MAFFT was further used to construct the phylogenetic tree. The maximum likelihood method was implemented in FastTree (Version 2.1.11) for constructing the phylogenetic tree [58]. The tree was visualized using FigTree (Version 1.4.4) (http://tree.bio.ed.ac.uk/software/figtree/). For modelling, Human GATD3A and DJ-1/PARK7 FASTA amino acid sequences were retrieved from UniProt database (https://www.uniprot.org), after which the sequences were submitted to Phyre2 (Protein Homology/analogY Recognition Engine V 2.0, (http://www.sbg.bio.ic.ac.uk/~phyre2/html/page.cgi?id=index). Results were ranked based on confidence score and percentage homology between GATD3A and proteins for which there exists crystal structures. Output files were visualized using PyMOL 2.4.2.

### Gatd3a^−/−^.B6 and Gatd3a^FLAG/FLAG^.B6 mice

All experiments were conducted according to protocols approved by a local Institutional Animal Care and Use Committee at the National Institutes of Health, Bethesda, MD. Insertion of a FLAG epitope tag (DYKDDDDK) to the C-terminus of GATD3A was achieved through CRISPR-mediated homologous recombination (HR) with a 151mer single-strand DNA oligo as the donor template (sequence in table below) as described elsewhere [24]. CRISPRscan was used to select guide RNA (gRNA) candidates near the target. Selected candidate gRNAs were synthesized by in vitro transcription and further tested by Surveyor assay in a MEF cell line carrying a Tet-On Cas9 expression cassette. Zygotes from C57BL/6J mice were microinjected with the final gRNA and the 151 bp donor with Cas9 protein, then transferred into a pseudopregnant CD1 surrogate mother (25 embryos per mother). F0 founders with correct HR were selected by PCR assay designed to amplify HR junctions from tail genomic DNA. PCR genotyping was performed using Standard Taq (New England Biolabs), with a forward primer (GATD3AFLAGFW) in Exon 7, and the reverse (GATD3AFLAGRV) in the 3′UTR of *Gatd3a*. Samples were resolved on a 4% 3:1 agarose gel to identify PCR products containing the FLAG epitope sequence (421 bp) versus a wildtype allele (397 bp). Founders were backcrossed onto C57BL/6J background for several generations.

For generating the *Gatd3a*^−/−^ mouse, two gRNAs flanking the genomic region of exon 1 to exon 3 (GATD3AKOE1 gRNA, GATD3AKOE3 gRNA) of the mouse *Gatd3a* gene were designed and generated as described above. A deletion allele, lacking 865 base pairs in one of the F0 founders, results in the loss of essential coding and splice sites, generating a truncated transcript containing a premature stop codon. PCR genotyping was performed using forward (GATD3AKOFW) and reverse (GATD3AKORV) primers flanking the gRNA cut sites. PCR using standard conditions (New England Biolabs) and resolution on a 1% agarose gel results in detection of deletion product of 213 bp and a wildtype allele of 1078 bp.

### Molecular cloning and qPCR analysis

HEK293 and COS7 cells were obtained from American Type Culture Collection and cultured at 37 °C in 5% CO_2_ in Dulbecco’s modified Eagle’s medium (Thermo Fisher Scientific) supplemented with 10% fetal bovine serum (Atlanta Biologicals) and 1% penicillin/streptomycin (Thermo Fisher Scientific). Cells were passaged at 75–80% confluency twice weekly.

RNA was isolated from HEK293 cells using TrizolLS according to the manufacturer’s instructions (Thermo Fisher Scientific). Complementary DNA (cDNA) was synthesized from 2 μg total RNA using Superscript II Reverse Transcriptase (Invitrogen) with Oligo(dT)_12-18_ according to the manufacturer’s instructions. Fragments of interest were PCR amplified from cDNA using high-fidelity Phusion Taq polymerase (New England Biolabs) with appropriate primers (below) including desired restriction sites and overhangs. Purified products and CMV vector were digested with appropriate restriction enzymes, and fragments were ligated using T4 DNA Ligase according to the manufacturer’s protocol (New England Biolabs). Positive clones were selected for in transformed DH5α *E. coli* using Kanamycin, and sequence verified. The Q5 site-directed mutagenesis kit was used for the generation of *p*CMV-Gatd3a^Δ1-33^-FLAG following the manufacturer’s guidelines (New England Biolabs).

For qPCR, cDNA was prepared as above from HEK293 cell RNA. Oligonucleotides used are listed in the table below. A QuantStudio 3 real-time PCR system (Thermo Fisher Scientific) was used to perform qPCR using PowerUp SYBR Green master mix, with samples ran in quadruplicate. The delta-delta CT method was used for quantification. Samples were normalized to *HPRT* house-keeping gene. To analyze mitochondrial biogenesis, qPCR was used to measure mitochondrial DNA to nuclear DNA ratios in MEFs, using the protocol outlined in [59].

**Table:** 

GATD3A FLAG 151mer	TAAAGAATGTGCTGGAACTCACGGGAAAGGACTACAAAGACGATGACGACAAGTAATGCCACCCGGACCACGCTTGGCCTCCGTGACTGTGGCATCCCCAGCCGGCGTGTGCTCCATGGCTTCAGCCCGGACACAGGAGCCGCTTAGCTAC
GATD3AE7 gRNA	GGGAAAGTAATGCCACC
GATD3AFLAGFW	TAAAGCCAGTGCCGACTTGT
GATD3AFLAGRV	CTCCTTCAGGCAGGAAGTGTCTAT
GATD3AKOE1 gRNA	CCGGGCTCCCTCCCAGCG
GATD3AKOE3 gRNA	CCGGGCTCCCTCCCAGCG
GATD3AKOFW	GCACGTTCTCCTGACACCACTGTG
GATD3AKORV	CAGCCGGATGACAGTTGCTTTGA
CMVGATD3AFW	CGCAGGTACCATGGCGGCTGTGAGGGCC
CMVGATD3ARV	TGCAGCGGCCGCTTATTACTTGTCGTCATCGTCTTTGTAGTCCCCCCCCCCCCCCCCCCCCTTTCCAGTGAGTTCCAGC
CMVGATD3AMLSFW	CTTCACCTCTCCGTGCCG
CMVGATD3AMLSRV	CATGGTACCGTCGACTGC
qGATD3A_FW	AAAACCTGAGCACGTTTGCC
qGATD3A_RV	TTCAGGACACGCTCCACTTC
qPPARGC1A_FW	GTCGGAAGACACCCTCTTCTC
qPPARGC1A_RV	CAGTCCAGGGGCAGAAAAGT
qESRRA_FW	CGGAGCCCCAGGTGAC
qESRRA_RV	TCTGTCTCCGAGGAACCCTT
qTFAM_FW	CCAAAAAGACCTCGTTCAGCTT
qTFAM_RV	CTTCAGCTTTTCCTGCGGTG
qHPRT_FW	ACCCCACGAAGTGTTGGATA
qHPRT_RV	AAGCAGATGGCCACAGAACT
PETDJ1FW	CGCAGCTAGCATGGCTTCCAAAAGAGCT
PETDJ1RV	TGCAGGATCCTTATTACTAGTCTTTAAGAACAAG
PETDJ1C106AFW	AGCCGCCATCGCTGCAGGTCCTACTG
PETDJ1C106ARV	ATCAGGCCCTTCCGGTTT
PETGATD3AFW	CGCAGGATCCATGATGCTTCACCTCTCCGTGCCG
PETGATD3ARV	TGCAAAGCTTTTATTACTTTCCAGTGAGTTCCAGC
PETGATD3AC176AFW	CATCGGCTTGGCGTGCATTGCACCTGTCCTCGCG
PETGATD3AC176ARV	GGCTTCCCGGCCTGGTGG
16s-rRNAFW	CCGCAAGGGAAAGATGAAAGAC
16s-rRNARV	TCGTTTGGTTTCGGGGTTTC
ND1FW	CTAGCAGAAACAAACCGGGC
ND1RV	CCGGCTGCGTATTCTACGTT
HK2FW	GCCAGCCTCTCCTGATTTTAGTGT
HK2RV	GGGAACACAAAAGACCTCTTCTGG

### Immunoblotting

For immunoblotting, cells were washed once in sterile 1× PBS, scraped, and transferred to a 1.5-mL Eppendorf, and collected by centrifugation. Cells were lysed in radioimmunoprecipitation assay (RIPA) buffer (50 mM Tris-HCl, pH 7.4, 150 mM NaCl, 1% NP-40, 1 mM EDTA, 1 mM EGTA, 0.1% SDS, 0.5% sodium deoxycholate, supplemented with PhosSTOP phosphatase inhibitors and cOmplete protease inhibitor cocktail (Millipore Sigma)) on ice for 30 min, followed by centrifugation (13,600×*g*, 15 min, 4 °C). Protein concentration was quantified using the Pierce BCA protein assay kit (Thermo Fisher Scientific). Samples were diluted to 1 μg/μl in Laemmli sample buffer with 2-mercaptoethanol (355 mM) and denatured for 10 min at 95 °C. In total, 25 μg of sample was loaded to a MiniPROTEAN TGX gel (Bio-Rad Laboratories) and subjected to SDS-PAGE. Protein was blotted to PVDF membrane using the TransBlot Turbo Transfer system (Bio-Rad Laboratories) and blocked using 5% milk dissolved in TBST (1× Tris buffered saline, pH 7.4, 0.1% Tween-20) for 1 h at RT. Membranes were probed with primary antibody diluted in 1% milk in TBST overnight at 4 °C, followed by 3 × 10-min washes in TBST, and subsequent probing with secondary HRP-conjugated antibody for 1 h at RT. Membranes were washed 3 × 10 min in TBST and imaged using Pierce ECL Western Blotting Substrate (Thermo Fisher Scientific) using a ChemiDoc Imaging System (Bio-Rad Laboratories). The following antibodies were used in this study:

**Table:** 

Antibodies		
Mouse Anti-FLAG M2	Millipore Sigma	Cat#F1804
Rabbit Anti-GRP75 H-155	SCBT	Cat#sc-13967
Mouse Anti-ATP5A 15H4C4	AbCam	Cat#ab14748
Rabbit Anti-Penta His	Qiagen	Cat#34660
Mouse Anti-Methylglyoxal (1,2-dicarbonyl)	Cell Biolabs Inc.	Cat#STA-011
Rabbit Anti-AGE	AbCam	Cat#ab23722
Rabbit Anti-FLAG M2	CST	Cat#2368
Rabbit Anti-Tomm20 FL-145	SCBT	Cat#sc-11415
Rabbit Anti-Cytochrome C D18C7	CST	Cat#11940
Rabbit Anti-TUFM	Thermo Fisher	Cat#PA5-27511
Rabbit Anti-DJ-1	AbCam	Cat#ab18257
Mouse Anti-GAPDH-71.1	Millipore Sigma	Cat#G8795
Mouse Anti-Total OXPHOS Cocktail	AbCam	Cat#ab110411
Rabbit Anti-C21orf33/GATD3A EPR13213	AbCam	Cat#ab181366
Rabbit Anti-GLO1	AbCam	Cat#ab96032
Rabbit Anti-GLO2	AbCam	Cat#ab154108
Rabbit Anti-Histone H3	AbCam	Cat#ab16056
Rabbit Anti-COXIV	AbCam	Cat#ab202554
Donkey Anti-Mouse IgG Alexa 488	Invitrogen	Cat#A21202
Donkey Anti-Rabbit IgG Alexa 568	Invitrogen	Cat#D1306
Goat Anti-Mouse IgG Alexa 594	Invitrogen	Cat#A11020
Goat Anti-Rabbit IgG ATTO647N	Rockland	Cat#611-156-122
Donkey Anti-Rabbit IgG HRP	Jackson Immunoresearch	Cat#711035152
Donkey Anti-Mouse IgG HRP	Jackson Immunoresearch	Cat#711035140

### Immunofluorescence, confocal, and STED microscopy

Cells were seeded in 24-well plate format on poly-D lysine-coated coverslips at 5 × 10^4^ cells/well. At 70–80% confluency, cells were transfected with 50 μl of OptiMEM (Thermo Fisher Scientific) containing a DNA:Lipofectamine 2000 complex in a ratio of 300 ng: 1 μl respectively, and incubated with complexes for 24–48 h, then fixed in 4% PFA for 5 min. For MitoTracker staining, cells were incubated with 250 nM Orange CMTMRos at 37 °C for 30 min, according to the manufacturer’s instructions (Invitrogen), prior to fixation. Samples were blocked using 0.22 μm sterile filtered blocking buffer; 10% normal donkey serum in PBS-T (1× phosphate-buffered saline, pH 7.4, containing 0.03% Triton X-100, and 0.002% NaN_3_) for 1 h at room temperature (RT). Samples were incubated with primary antibody (FLAG (Millipore Sigma, F1804, 1:1000) mtHsp70/GRP75 H-155 (SCBT, sc-13967, 1:250), TOMM20 (SCBT, sc-11415, 1:200) diluted in blocking buffer overnight at 4 °C, followed by 3 × 10 min washes in PBS-T, and incubated with secondary antibodies (anti-Mouse IgG Alexa 488, Invitrogen, A21202, 1:500, and anti-Rabbit IgG, Alexa 568, A10042, 1:500) and counterstain DAPI (4',6-Diamidino-2-Phenylindole, Dihydrochloride, Invitrogen, D1306, 1 μg/ml) diluted in blocking buffer for 1 h at RT. Samples were washed 3 × 10 min in PBS-T and mounted with Fluoromount-G (Southern Biotech). Samples were imaged using a Zeiss LSM 880 confocal microscope operating ZEN image capture software. To analyze mitochondrial morphology in MEFs, the Mitochondria Analyzer plugin was used on confocal microscopy images using Mitotracker Orange [60]. Measurements from individual mitochondria were exported from Mitochondria Analyzer into Excel and Graphpad Prism. Mean mitochondrial measurements for each cell were compared between *Gatd3a*^+/+^ and *Gatd3a*^−/−^.

For super-resolution stimulated emission depletion (STED) microscopy, COS7 cells were transiently transfected with *p*CMV-Gatd3a-FLAG as described above, fixed in 4% PFA, blocked using blocking buffer (10% normal goat serum, 0.03% Triton X-100, 0.002% NaN_3_), and incubated with primary antibodies directed against the FLAG epitope (Millipore Sigma, F1804, 1:100), Tomm20 FL-145 (SCBT, sc-11415, 1:50) and mtHsp70/GRP75 H-155 (SCBT, sc-13967, 1:50), and secondary antibodies conjugated with Alexa 594 (Invitrogen, A-11020), and ATTO 647N (Rockland, 611-156-122), and mounted using ProLong Glass Antifade Mountant (Invitrogen). Time-gated STED images were obtained using a commercial STED microscope (SP8 STED 3X; Leica Microsystems), equipped with a white-light laser (470–670nm) and 592 nm, 660 nm, and a pulsed 775-nm STED depletion lasers. A 100×/1.4-N.A. oil immersion objective lens (HCX PL APO STED white; Leica Microsystems) was used for imaging. For resolution comparison, confocal and STED images were taken sequentially for Tomm20 and mtHsp70 labeled with ATTO 647N and imaged using 640-nm excitation and 650–720 nm emission range and for GATD3A:FLAG labeled with Alexa 594 and imaged using 560-nm excitation, a 575- to 630-nm emission detection range at a scan speed of 600 lines per second, with gated hybrid detectors, and the pulsed 775-nm STED depletion laser for both. Samples were imaged with a scan speed of 600 lines per second, a pixel size of 30 nm, and 6-line averages. Z-stacks were collected at 0.160-μm-depth intervals. Fluorescence intensity profiles along a line were measured using Leica LAS X software 3.5.6. (Leica Microsystems, Mannheim, Germany). Images were further deconvolved using the classical maximum likelihood estimation algorithm in Huygens Professional software version 19.10.0p2 64b (SVI, Hilversum, NL), examined, and reconstructed using Imaris software version 9.2.1 (Bitplane, Zurich, Switzerland).

### Recombinant protein production

Human DJ-1 and GATD3A, with its mitochondrial localization signal removed, were amplified from cDNA and cloned into the pET28 expression vector containing a 6X-His tag in the C-terminal, with T7 directed expression controlled by a lac operon. A C106A and C176A mutant was generated for DJ-1 and GATD3A respectively using the Q5 site-directed mutagenesis kit (New England Bio Labs). All oligonucleotides used for cloning are given in the supplemental table. BL21 DE3 cells were transformed, and positive clones selected with Kanamycin were induced with 1 mM IPTG for 4–5 h at 37 °C. Cells were pelleted and lysed in lysis buffer (50 mM phosphate buffer, pH 7.4, 300 mM NaCl, 10 mM Imidazole, 14.3 mM 2-mercaptoethanol, supplemented with 1 mg/ml lysozyme and protease inhibitors), on ice for 30 min, followed by sonication at 10% amplitude level (Misonix XL2000) using a microtip probe, for 10 s, repeated 6 times, and centrifuged at 10,000×*g* for 30 min at 4 °C. Samples were filtered (0.45 μm) before loading onto a HisTrap HP His tag protein purification column (GE Healthcare) on an ÄKTAexplorer FPLC system programmed using UNICORN manager. Total sample was loaded onto the affinity column at a flow rate of 0.5 ml/min and was washed with buffer containing 10 mM imidazole. Affinity-purified protein was eluted over a 10–100 mM imidazole gradient and collected in 1-ml aliquots using a Frac-950 fraction collector. Due to co-elution of bacterial proteins at this concentration of imidazole, size exclusion chromatography was used to further purify the recombinant protein. In total, 100 μl of purified protein was loaded using a loop onto a Superdex 75 Increase 10/300 GL size exclusion column and eluted in sterile filtered, degassed sodium phosphate buffer at a flow rate of 0.3 ml/min. All recombinant samples were analyzed using SDS-PAGE followed by Coomassie Brilliant Blue staining according to the manufacturer’s instructions (Thermo Fisher Scientific), and immunoblotting using a Penta-His antibody diluted in a modified blocking buffer of 3% BSA in TBS (Qiagen, 34660, 1:1000).

### Amidolysis and slot blot immunodetection assays

A modified version of the Glutamine/Glutamate-Glo Assay (Promega, J8021) was used to assess the amidolysis capacity of GATD3A and DJ-1, using L-glutamine as the substrate. An L-glutamine standard curve was prepared using a 100 mM L-glutamine stock (Thermo Fisher Scientific). Briefly, glutamine standards were aliquoted in triplicate per condition in a flat-bottomed white plastic 96-well plate. A recombinant protein sample [2 μM (GATD3A or DJ-1), glutaminase, and no enzyme control] was diluted in glutaminase buffer and added to wells containing glutamine, and gently mixed. Samples were incubated for 30 min at RT to allow for conversion of glutamine to glutamate. Glutamate detection solution (luciferin detection solution, reductase, reductase substrate, glutamate dehydrogenase, NAD) was prepared and added to each well resulting in a final ratio of 1:1:2 for sample volume:recombinant protein solution:glutamate detection solution. Following gentle shaking, the reaction was incubated in dark for 1 h at room temperature. Luminescence was detected on a GloMax Navigator Microplate Luminometer. Signal was detected for 1 s, where signal is directly proportional to the glutamate concentration.

For the deglycase assay, plasmid DNA (1 ng/μl) and γ-globulin (1 mg/ml) was incubated with either glyoxal (GO) or methylglyoxal (MGO) (5 mM) in N_2_ gassed sodium phosphate buffer (pH 7.4) at 37 °C. Timepoints were stored at – 20 °C. After 2 h, glycated samples were incubated with 5 μg of recombinant protein (DJ-1, GATD3A, or GATD3A^C176A^) for a further 2 h at 37 °C. For immune-detection, DNA samples were denatured in SSC buffer at 98 °C for 10 min and applied to Amersham Hybond-N+ nylon membranes using a Minifold II vacuum slot blot manifold. DNA was crosslinked to nylon membranes on a Stratalinker UV Crosslinker at 120,000 μJ/cm^2^ (Stratagene). Protein samples were denatured in 8M urea at 30 °C for 10 min and applied similarly to nylon membranes. Membranes were blocked in 5% milk in TBST and incubated overnight in either antibodies with epitopes raised against either 1,2-dicarbonyls (Cell Biolabs Inc., STA-011, 1:1000) or advanced glycation end products (AbCam, ab23722, 1:1000). Blots were developed as previously described. For pulse-chase experiments, mouse embryonic fibroblasts were isolated from the *Gatd3a*^−*/*−^ mouse and wildtype littermates as previously described [61]. Cells were seeded (5 × 10^5^) in a 6-well plate and treated with 1 mM GO, and removed following 12 h of incubation. Samples were allowed to recover for a further 12 h before harvesting in RIPA buffer and were subjected to immunoblotting as described above.

### RNA immunoblotting

Mice were perfused with HBSS and heart left ventricle was isolated and macerated on ice. Samples were suspended in sterile PBS and subjected to RNA isolation using the miRNeasy kit, according to the manufacturer’s instructions (Qiagen). Concentration and RNA integrity was assessed using an Agilent RNA 6000 Nano kit. Purified RNA samples were normalized and denatured in 2× NorthernMax formaldehyde loading dye (Thermo Fisher Scientific) at 65 °C for 15 min. Samples were electrophoresed in 10% Novex TBE Urea gels at 250 V for 45 min. Gels were stained using UltraPure EtBr (1:10,000) in 0.5× TBE for 1 h before imaging using a UV transilluminator. Gels were destained in 0.5× TBE before electroblotting to a nylon membrane at 18V/~300 mA for 60 min using a semidry transfer apparatus, and UV crosslinked as described previously. Membranes were blocked in 5% milk and subjected to immunodetection using methods described above.

### Mitochondrial isolation and fractionation

HEK293 cells were lysed in 0.25 M sucrose buffer and cell debris was removed following centrifugation at 100×*g*. Supernatant was subjected to centrifugation at 800×*g* and nuclear pellet was retained. Supernatant was centrifuged at 8000×*g*, and post-mitochondrial supernatant was retained as a cytosolic fractionation following clearing at 15,000×*g.* SDS-PAGE was performed on protein samples as above and probed for nuclear (Histone H3, ab16056, 1:2000) cytosolic (GAPDH, Millipore Sigma G8795, 1:2000) and mitochondria (COXIV, ab202554, 1:2000). In mice, isolated mitochondria were fractionated using previously published methods [62] with minor adjustments. Briefly, *Gatd3a*^FLAG/FLAG^ mice were anaesthetized (Ketamine 80–100 mg/kg, Xylazine 8–10 mg/kg) and perfused using sterile HBSS (Thermo Fisher Scientific). Hearts were isolated, diced, and homogenized in a Teflon-glass Potter-Elvehjem homogenizer with ice-cold isolation buffer (220 mM mannitol, 700 mM sucrose, 2 mM Tris, pH 7.4, 1 mM EDTA, 20 mM HEPES, pH 7.2, 0.4% BSA). Samples were centrifuged at 600×*g* for 5 min. Supernatants were centrifuged at 17,000×*g* for 10 min at 4 °C. Pellets were washed twice in isolation buffer, without EDTA. Mitochondrial concentration was determined using a Bradford assay (Bio-Rad Laboratories). Mitochondria were incubated at 2 mg/ml in hypotonic buffer (2 mM KCl, 10 mM HEPES, pH 7.2) for 20 min on ice, and centrifuged at 10,000×*g*. Pellets were washed twice in wash buffer (150 mM KCl, 10 mM HEPES, pH 7.2). All supernatants were combined and subjected to TCA precipitation, creating an intermembrane space fraction. Pellets were resuspended in hypotonic buffer and subjected to three freeze thaw cycles, followed by sonication (1% amplitude, 5 s, ×3) and centrifuged at 110,000×*g* for 30 min and retained as an outer and inner membrane fraction. Supernatant was subjected to TCA precipitation and stored as a matrix fraction. Samples were subjected to SDS-PAGE and blotting. Membranes were probed for FLAG M2 antibody (CST, 2368, 1:1000) and compartment-specific antibodies; outer membrane (Tomm20 FL-145, SCBT, sc-11415, 1:1000), inner membrane (ATP5A 15H4C4, Abcam, ab14748, 1:2500), intermembrane space (Cytochrome c D18C7, CST, 11940, 1:3000), and matrix (mtHsp70/GRP75 H-155, SCBT, sc-13967, 1:500) and probed with HRP-conjugated secondary antibodies raised against rabbit IgG (Jackson ImmunoResearch, 711035152, 1:3000) and mouse IgG (Jackson ImmunoResearch, 711035140, 1:3000).

### Immunoprecipitation and mass spectrometry

GATD3A:FLAG was immunoprecipitated from *Gatd3a*^FLAG/FLAG^ mouse heart mitochondria using methods described previously [63] with the following modifications. Following mitochondria isolation described above, sample was resuspended in ice-cold IP buffer (20 mM HEPES, pH 7.4, 100 mM NaCl, 10% glycerol, 1 mM DTT, supplemented with protease inhibitors). Following maceration with a glass homogenizer, detergent solution was added (final concentration 2% Digitonin, 2.5% NP-40), and end over end agitation was carried out for 15 min at 4 °C, followed by centrifugation (16,000×*g*, 10 min, 4 °C). Supernatant concentration was quantified using the BCA method. Magnetic anti-FLAG epitope conjugated beads (Millipore Sigma, M8823) were washed with IP buffer containing detergent three times, and equal concentrations of protein were added to beads at 4 °C overnight. Beads were washed (20 mM HEPES, pH 7.4, 100 mM NaCl, 0.05% digitonin, 10% glycerol) four times, with the final wash containing no detergent or glycerol. Protein was eluted in final wash buffer containing 0.2 mg/ml FLAG peptide. Eluate was subjected to centrifugal filtration for removal of FLAG peptide using 3K NMWL filtration units, using final wash buffer for dialysis.

Immunoprecipitated proteins were dried and solubilized with 6 M urea, 2 M thiourea, and 50 mM ammonium bicarbonate, pH 7.8. Protein solutions were reduced with 10 mM dithiothreitol (DTT) to reduce the disulfide bonds for 1 h at 37 °C. To prevent reformation of disulfide bonds, samples were incubated at room temperature in the dark with 20 mM of iodoacetamide for 45 min. An additional 10 mM of DTT was added to quench the excess iodoacetamide for 10 min at room temperature. All samples were diluted 6-fold with 50 mM ammonium bicarbonate followed by the addition of Trypsin (Promega, Madison, WI, USA) for overnight digestion at 37 °C. Peptide digests were acidified with 10% trifluoroacetic acid (TFA) to a final 1% concentration, pH 3. Digests were dried down and resuspended in 0.1% formic acid. The resulting peptide mixture was concentrated and desalted with C18 Zip-tips (Millipore Sigma), following the company’s protocol. Desalted digests were analyzed by mass spectrometry. Mass spectrometry experiments were performed on an Orbitrap Lumos Tribrid coupled with an Ultimate 3000-nLC (Thermo Fisher Scientific). Peptides were separated on an EASY-Spray C18 column (Thermo Scientific; 75 μm × 50 cm inner diameter, 2 μm particle size, and 100 Å pore size). Separation was achieved by 4–28% linear gradient of acetonitrile + 0.1% formic acid over 60 min. An electrospray voltage of 1.9 kV was applied to the eluent via the EASY-Spray column electrode. The Orbitrap Lumos was operated in positive ion data-dependent mode. Full-scan MS1 was performed in the Orbitrap with a normal precursor mass range of 375–1500 m/z at a resolution of 120k. The automatic gain control (AGC) target and maximum accumulation time settings were set to 4 × 105 and 50 ms, respectively. MS2 was triggered by selecting the most intense precursor ions above an intensity threshold of 5 × 103 for collision-induced dissociation (CID)-MS2 fragmentation with an AGC target and maximum accumulation time settings of 2 × 103 and 300 ms, respectively. Mass filtering was performed by the quadrupole with 1.6 m/z transmission window, followed by CID fragmentation in the ion trap (rapid mode) and a normalized collision energy (NCE) of 35%. To improve the spectral acquisition rate, parallelizable time was activated. The number of MS2 spectra acquired between full scans was restricted to a duty cycle of 3 s. Raw data files were processed using Proteome Discoverer (v2.2, Thermo Fisher Scientific), using Mascot v2.5.1 (Matrix Sciences) search node. All searches were performed against UniProt Knowledgebase protein database with the selected taxonomy Mus musculus (mouse). Search modifications used were as follows: (fixed) carbamidomethyl of cysteine, (variable) oxidation of methionine, deamidation (NQ), and acetyl on protein N-terminus. For MS2, the precursor mass tolerance of 12 ppm and fragment mass tolerances of 0.5 Da were applied, respectively. Up to two missed tryptic cleavages were permitted. Percolator was used to calculate the false discovery rate (FDR) of peptide spectrum matches, set to a *p*-value of 0.05 [64].

For identification of co-eluting proteins following FPLC, samples were run on SDS-PAGE and stained using Coomassie Brilliant Blue. Bands of interest were excised, and gel pieces were destained using destaining solution (10% glacial acetic acid, 50% methanol, 40% Milli-Q H_2_O). The excised gel bands were subjected to previously published trypsin digestion methods with minor modifications [65]. Post reduction and alkylation steps, the dried gel bands were rehydrated in a 12.5 ng/μL trypsin solution in 50 mM ammonium bicarbonate/0.05% AALS (Anionic Acid Labile Surfactant, Protea), overnight digestion at 37 °C, pH 7.8. Digests were collected, acidified with 10% trifluoroacetic acid (TFA) to a final concentration of 1%, agitating for 15 min at 37°C for surfactant degradation and dried down. The resulting peptide mixture was concentrated and desalted with C18 Zip-tips (Millipore Sigma), following the company’s protocol. Peptides were separated on an EASY-Spray C18 column (Thermo Scientific; 75 μm × 50 cm inner diameter, 2 μm particle size and 100 Å pore size). Separation was achieved by 4–30% linear gradient of acetonitrile + 0.1% formic acid for 65 min. An electrospray voltage of 1.9 kV was applied to the eluent via the EASY-Spray column electrode. The Orbitrap Lumos was operated in positive ion data-dependent mode. Full-scan MS1 was performed in the Orbitrap with a normal precursor mass range of 375–1500 m/z at a resolution of 120k. The automatic gain control (AGC) target and maximum accumulation time settings were set to 4 × 105 and 50 ms, respectively. MS2 was triggered by selecting the most intense precursor ions above an intensity threshold of 2.5 × 104 for collision-induced dissociation (CID)-MS2 fragmentation with an AGC target and maximum accumulation time settings of 5 × 104 and 50 ms, respectively. Mass filtering was performed by the quadrupole with 0.7 m/z transmission window, followed by CID fragmentation in the Orbitrap and a collision energy of 35% at a resolution of 15k. To improve the spectral acquisition rate, parallelizable time was activated. The number of MS2 spectra acquired between full scans was restricted to a duty cycle of 3 s. Raw data files were processed with Proteome Discoverer (v2.2, Thermo Fisher Scientific) software, using Sequest HT (Thermo Fisher Scientific) search node. All peak lists were searched in parallel against the UniProtKB/Swiss-Prot protein databases Homo sapiens (20,316 sequences, released 2018_11) and *Escherichia coli* strain K12 (4418 sequences, released 2018_11) concatenated with reversed copies of all sequences. The following search parameters were set for MS1 tolerance of 10 ppm; orbitrap-detected MS/MS mass tolerance of 0.02 Da; enzyme specificity was set as trypsin with maximum two missed cleavages; minimum peptide length of 6 amino acids; fixed modification of Cys (carbamidomethylation); variable modifications of methionine oxidation, deamidation of Asn, and Gln, and Protein N-terminus Acetyl. Target Decoy was used to calculate the false discovery rate (FDR) of peptide spectrum matches, set to a *p*-value < 0.05 [66].

### Oxygen Consumption Rate (OCR) measurement

MEFs were cultured in DMEM + 10% FBS. Cells were collected by trypsinization, counted, and plated (20,000 cells per well) on Seahorse XFe24 multiwell plates. Plates were incubated at 37 °C overnight in a CO_2_ incubator. The following day, medium was removed, cells were washed with DMEM without FBS, and 1 ml of DMEM containing 0.2 mM glyoxal was added to the experimental wells. We then added 1 ml medium without the compound to the control wells, and the plates were incubated at 37 °C overnight. After treatment, the cells were washed with PBS, then with the Seahorse XF DMEM buffer, and OCR was measured using the XFe24 Extracellular Flux Analyzer (Agilent Technologies). Inhibitors were used at the following concentrations: oligomycin, 2 μM; Bam15, 5 μM; and rotenone, 1 μM. The results were analyzed using the Agilent Wave software. Results were normalized to cell number.

### Transmission Electron Microscopy (TEM)

Hearts from perfused mice were isolated, and 1-mm^3^ sections of left ventricle were dissected and fixed 2.5% buffered glutaraldehyde. Tissues were processed for TEM as previously described [67]. Briefly, specimens were washed in phosphate-buffered saline (PBS), post-fixed in 0.5% osmium tetroxide (OsO4), rinsed, dehydrated, then embedded in epoxy resin. The blocks were sectioned at ~90-nm thickness on a Leica EM UC6 ultramicrotome (Leica, Austria), double-stained with uranyl acetate and lead citrate, and imaged with JEOL JEM-1010 electron microscope (JEOL, Japan).

Mitochondrial electron density was quantified using the histogram function in Fiji (ImageJ). *N* = 4 left ventricles per genotype were used, with *N* = 3 images per animal being used for quantification. Electron density is represented as a ratio of the mean greyscale area for three muscle fiber sections compared to the mean greyscale for all mitochondria in a field of view.

### Quantification and statistical analysis

Two-tailed *t* tests were used for comparison between two groups using GraphPad Prism v8.0. All comparisons were two-sided, and *p*-values of less than 0.05 were considered to indicate statistical significance. In the instances where alternative statistical tests were used, this has been noted in the figure legend. Specific statistical tests and metrics (median, mean, standard error) used for comparisons, along with sample sizes, are described in the figure legends.

## Supplementary Information

**Additional file 1: Fig. S1.** Recombinant protein production, immunoreactivity to 1,2-dicarbonyls and AGEs. **Fig. S2.** Protein interaction network reveals heterogeneity in GATD3A interactors. **Fig. S3.** Loss of mitochondrial GATD3A is not compensated for by other dicarbonyl defense enzymes. **Fig. S4.** Overexpression of GATD3A increases mitochondrial fragmentation.

**Additional file 2.** Uncropped gel images with indicated figures.

## Data Availability

All materials will be available from the corresponding author upon request and per NIH policy. All mass spectrometry datasets generated from this research are available through the PRIDE consortium with accessions: PXD030977 [68] and PXD030978 [69]. The other datasets analyzed during the current study were from the Genotype-Tissue Expression Project, dbGaP Study Accession: phs000424.v7.p2 [16, 70] and NCBI GEO accession GSE115828 [17, 71]. This study used publicly available coding software for analysis of datasets.

## Abbreviations

AGE: Advanced glycation end product
CRISPR: Clustered regularly interspaced short palindromic repeats
DJ-1/PARK7: Parkinsonism-associated deglycase
GATD3A: Glutamine amidotransferase-like class 1 domain-containing 3A
GO: Glyoxal
gRNA: Guide RNA
HR: Homologous recombination
MEF: Mouse embryonic fibroblast
MGO: Methylglyoxal
MLS: Mitochondrial localization signal
mt-mRNA: Mitochondrial messenger RNA
NECM: Non-enzymatic chemical modification
STED: Super-resolution stimulated emission depletion microscopy
TEM: Transmission electron microscopy
UTR: Untranslated region
