# Supplementary material for: GATD3A, a mitochondrial deglycase with evolutionary origins from gammaproteobacteria, restricts the formation of advanced glycation end products
Source: BMC Biol. 2022 Mar 21;20:68. doi: 10.1186/s12915-022-01267-6 (PMC8935817; doi:10.1186/s12915-022-01267-6)
Supplement: Supplementary file 1 — Additional file 1: Fig. S1. Recombinant protein production, immunoreactivity to 1,2-dicarbonyls and AGEs. Fig. S2. Protein interaction network reveals heterogeneity in GATD3A interactors. Fig. S3. Loss of mitochondrial GATD3A is not compensated for by other dicarbonyl defense enzymes. Fig. S4. Overexpression of GATD3A increases mitochondrial fragmentation. [file 12915_2022_1267_MOESM1_ESM.docx]

**ADDITIONAL FILE 1**

**GATD3A, a mitochondrial deglycase with evolutionary origins from gammaproteobacteria, restricts the formation of advanced glycation endproducts**

Andrew J. Smith^1,4^, Jayshree Advani^1^, Daniel C. Brock^1^, Jacob Nellissery^1^, Jessica Gumerson^1^, Lijin Dong^2^, L. Aravind^3^, Breandán Kennedy^4^, Anand Swaroop^1^*

^1^Neurobiology, Neurodegeneration and Repair Laboratory, National Eye Institute, National Institutes of Health, MSC0610, 6 Center Drive, Bethesda, MD 20892, USA.

^2^Genome Engineering Core, National Eye Institute, National Institutes of Health, 6 Center Drive, Bethesda, MD 20892, USA.

^3^National Center for Biotechnology Information, National Library of Medicine, National Institutes of Health, Bethesda, MD 20894, USA.

^4^UCD School of Biomolecular and Biomedical Science, Conway Institute, University College Dublin, Belfield, D4, Dublin, Ireland.

Andrew J. Smith [andrew.james.smith.1@gmail.com](mailto:andrew.james.smith.1@gmail.com)

Jayshree Advani [jayshree.advani@nih.gov](mailto:jayshree.advani@nih.gov)

Daniel C. Brock [daniel.brock@nih.gov](mailto:daniel.brock@nih.gov)

Jacob Nellissery [nellisseryj@nei.nih.gov](mailto:nellisseryj@nei.nih.gov)

Jessica Gumerson [jessica.gumerson@nih.gov](mailto:jessica.gumerson@nih.gov)

Lijin Dong [dongl@nei.nih.gov](mailto:dongl@nei.nih.gov)

L. Aravind [aravind@ncbi.nlm.nih.gov](mailto:aravind@ncbi.nlm.nih.gov)

Breandán Kennedy [brendan.kennedy@ucd.ie](mailto:brendan.kennedy@ucd.ie)

Anand Swaroop [swaroopa@nei.nih.gov](mailto:swaroopa@nei.nih.gov)

* Corresponding author

**Table of Contents for Additional File 1**

**Includes Figures S1-S4, Supporting data for main Figures 1-5**

Fig. S1. Recombinant protein production, immunoreactivity to 1,2-dicarbonyls and AGEs.

Fig. S2. Protein interaction network reveals heterogeneity in GATD3A interactors.

Fig. S3. Loss of mitochondrial GATD3A is not compensated for by other dicarbonyl defense enzymes.

Fig. S4. Overexpression of GATD3A increases mitochondrial fragmentation.

**
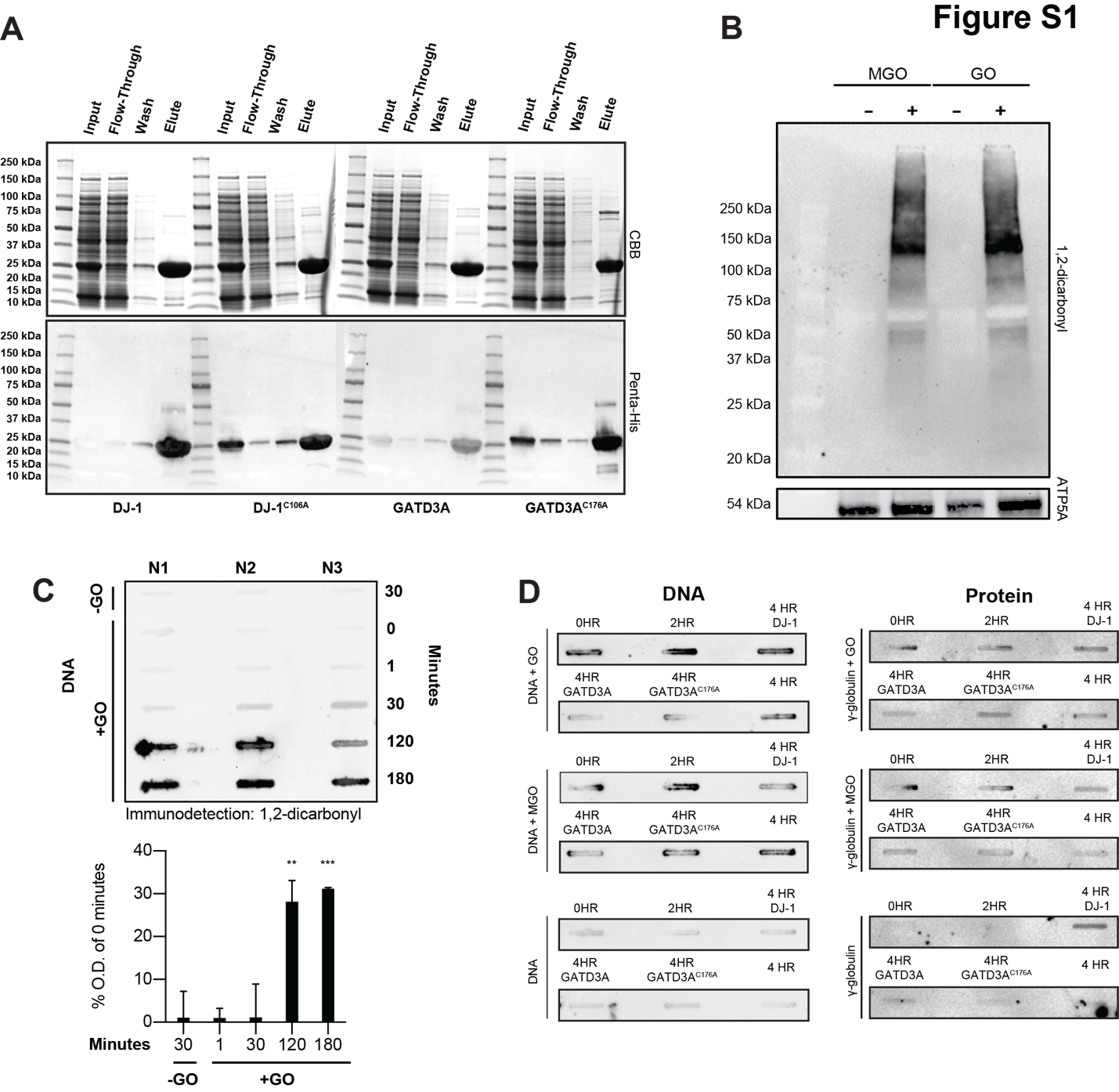
**

**Fig. S1. Recombinant protein production, immunoreactivity to 1,2-dicarbonyls and AGEs.**

**A:** Recombinant protein quality control of DJ-1, DJ-1 C106A, GATD3A, and GATD3A C176A proteins expressed in E. coli. Coomassie brilliant blue (CBB) staining displays total protein and the purity of eluted fractions (upper panel). Immunodetection of 6X-His tagged proteins using penta-His antibody (lower panel) reveals efficient purification of recombinant proteins. **B:** A commercial monoclonal antibody raised against BSA-conjugated methylglyoxal shows immunoreactivity of HEK293 cells untreated (-) or treated (+) with 5 mM of either 1,2-dicarbonyl methylglyoxal (MGO) and glyoxal (GO). Treatment with MGO or GO results in protein cross-linking as demonstrated by enhanced immunodetection of high molecular mass species. ATP5A serves as a loading control for all samples. **C:** 1-2 dicarbonyl immunoreactivity of DNA incubated with glyoxal (GO) increases over time (minutes). Quantification of blot was performed comparing O.D. to the 0-minute timepoint. (p<0.05, two-tailed student’s T-test, N=3, error bars = SEM). **D:** Slot blot immunodetection assays. Incubation of glycated plasmid DNA and γ-globulin protein with recombinant DJ-1 or GATD3A protein prevents the formation of AGEs in vitro. Abrogation of catalytic cysteine residue in GATD3A augments its deglycase activity. Quantification for samples is shown in main Fig. 2D.

**
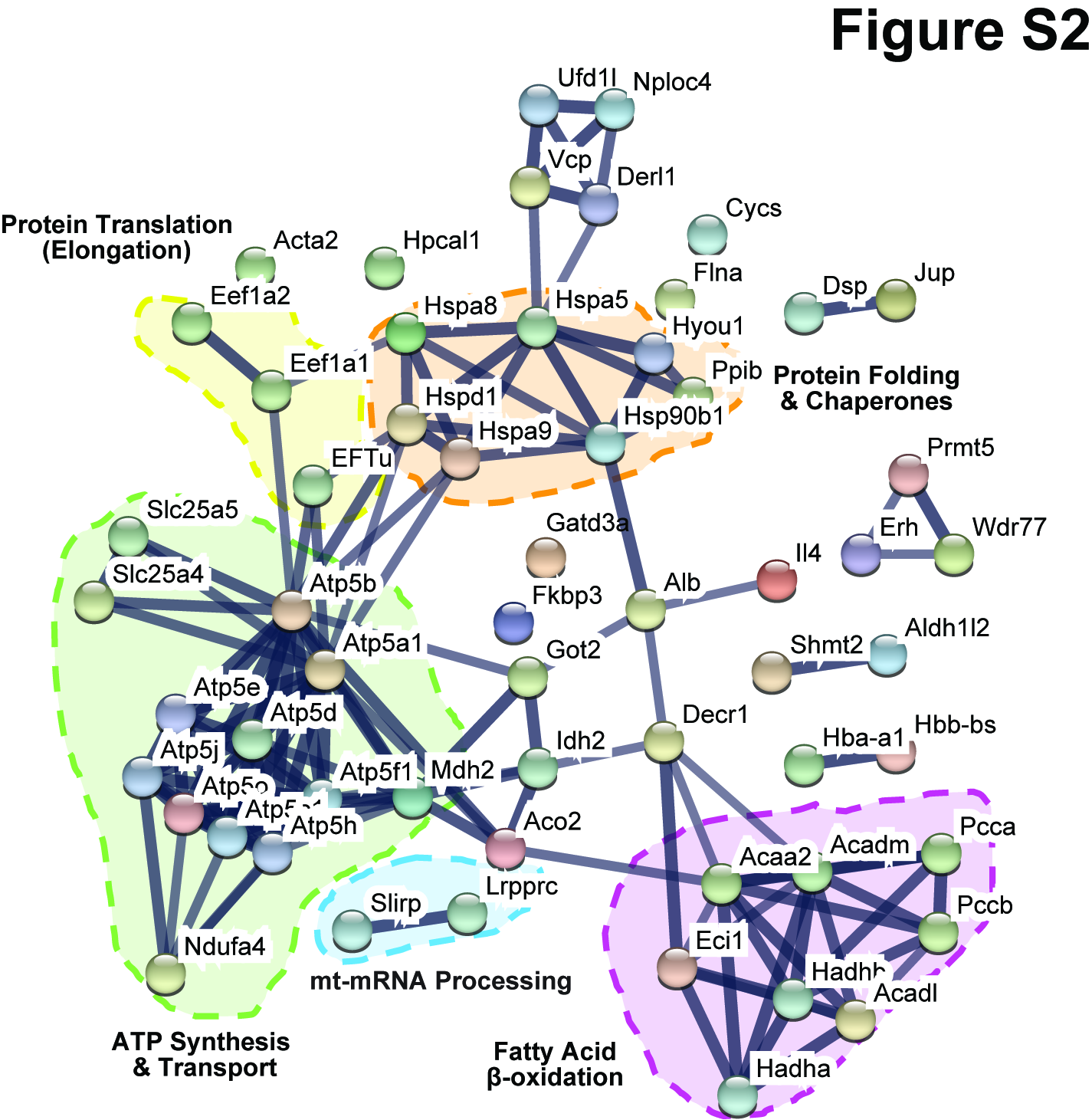
**

**Fig. S2. Protein interaction network reveals heterogeneity in GATD3A interactors**

Protein interaction network of GATD3A-FLAG co-immunoprecipitated factors. Proteins with

peptide spectral matches (PSM) >10 in three replicate experiments were used for generating the network. Heterogenous mitochondrial specific pathways were identified in the analysis.

**
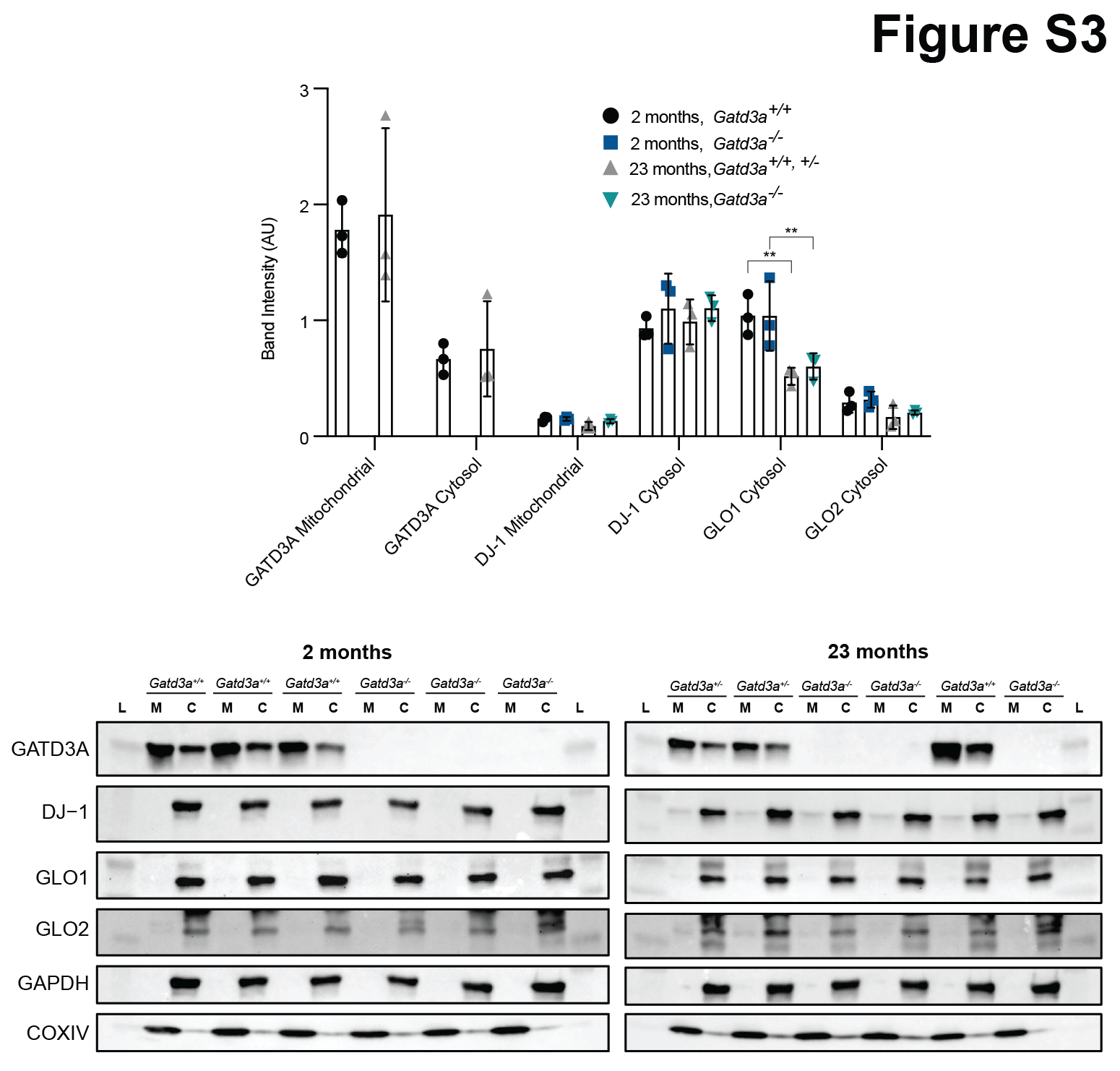
**

**Fig. S3. Loss of mitochondrial GATD3A is not compensated for by other dicarbonyl defense enzymes.**

Quantification of dicarbonyl defense enzymes in *Gatd3a*^+/+^, *Gatd3a*^+/-^, *and Gatd3a*^+/-^ mouse heart left ventricle at 2 months and 23 months of age (N=3/genotype, *p* = **<0.01, Student’s two-tailed T-test). Western blots were performed on mitochondrial enriched samples (M), and post-mitochondrial cytosolic supernatant (C) probed for GATD3A, DJ-1, GLO-1 and GLO-2. GAPDH and COXIV served as cytosolic and mitochondrial controls, respectively.

**
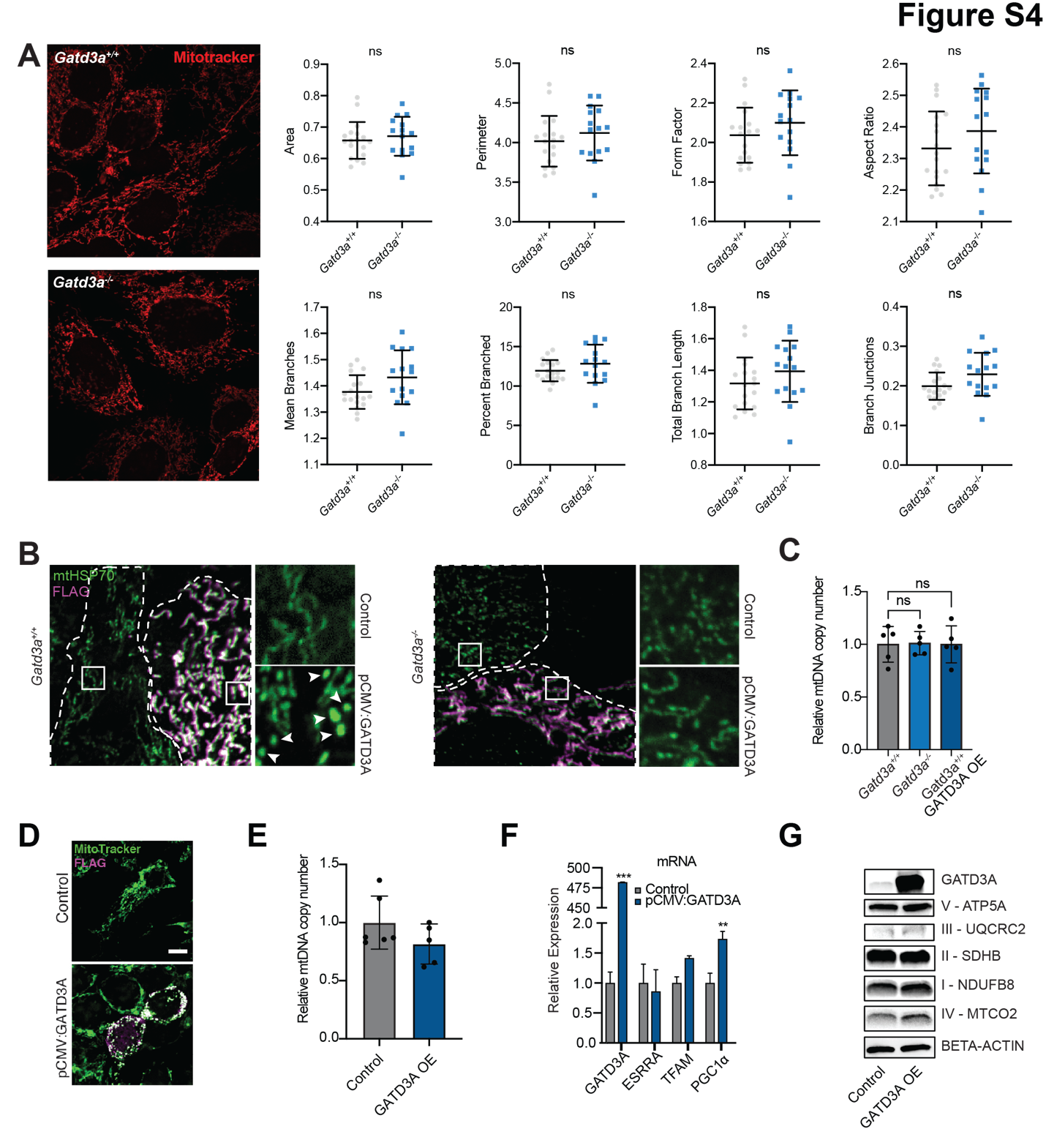
**

**Fig. S4. Overexpression of GATD3A increases mitochondrial fragmentation.**

**A:** Extended data of Fig. 5C demonstrating no significant difference in mitochondrial morphology or connectivity between *Gatd3a*^+/+^ *and Gatd3a*^+/-^ MEFs (N=21 per genotype, two-tailed Welch’s t-test, lines represent mean value. **B:** Mitochondrial morphology appears altered by overexpression of GATD3A in wildtype (left panel) but is unaffected in *Gatd3a^-/-^* (right panel) MEFs. Overexpression of GATD3A in wildtype MEFs increased apparent mitochondrial size compared to control. **C:** mitochondrial DNA (mtDNA): nuclear DNA ratio is unchanged in *Gatd3a^-/-^* MEFs, and *Gatd3a^+/+^* MEFs overexpressing GATD3A compared to *Gatd3a^+/+^* MEFs. **D**: Qualitative observance of mitochondrial fragmentation following overexpression of GATD3A in HEK 293 cells, similar to observed phenotype in MEFs (Fig. S3A) (scale = 10 μm, representative image of N=6). **E:** Overexpression of GATD3A in HEK 293 cells does not affect mtDNA copy number relative to nuclear DNA**. F**: Enhanced mRNA expression of *PGC1α*, the master regulator of mitochondrial biogenesis, by GATD3A over-expression. The data was normalized to *HPRT* expression. (two-tailed Student’s t-test was performed comparing control transfected HEK293 to *p*CMV:GATD3A transfected cells. p<0.05). **G:** Protein expression of ETC and OXPHOS components in HEK 293 cells overexpressing GATD3A:FLAG.
