# Supplementary material for: GATD3A, a mitochondrial deglycase with evolutionary origins from gammaproteobacteria, restricts the formation of advanced glycation end products
Source: BMC Biol. 2022 Mar 21;20:68. doi: 10.1186/s12915-022-01267-6 (PMC8935817; doi:10.1186/s12915-022-01267-6)

ADDITIONAL FILE 2

GATD3A, a mitochondrial deglycase with evolutionary origins from gammaproteobacteria, restricts the formation of advanced glycation endproducts

Andrew J. Smith[1,4], Jayshree Advani[1], Daniel C. Brock[1], Jacob Nellissery[1], Jessica Gumerson[1], Lijin Dong[2], L. Aravind[3], Breandán Kennedy[4], Anand Swaroop[1]*

[1]Neurobiology, Neurodegeneration and Repair Laboratory, National Eye Institute, National Institutes of Health, MSC0610, 6 Center Drive, Bethesda, MD 20892, USA.
[2]Genome Engineering Core, National Eye Institute, National Institutes of Health, 6 Center Drive, Bethesda, MD 20892, USA.
[3]National Center for Biotechnology Information, National Library of Medicine, National Institutes of Health, Bethesda, MD 20894, USA.
[4]UCD School of Biomolecular and Biomedical Science, Conway Institute, University College Dublin, Belfield, D4, Dublin, Ireland.

This file includes uncropped gel images in figures as indicated.

Fig 2A

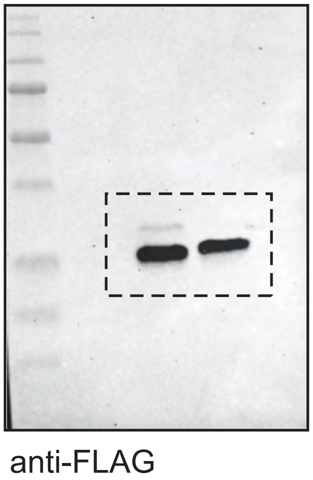

Fig 2B

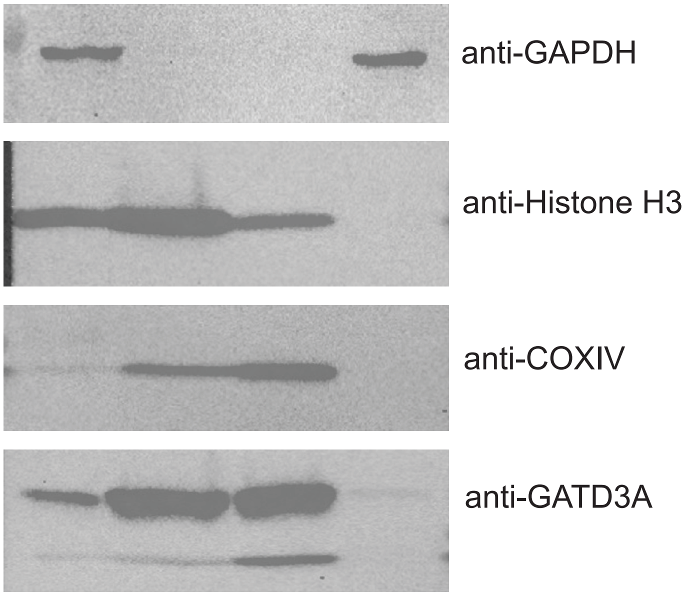

Fig 2C

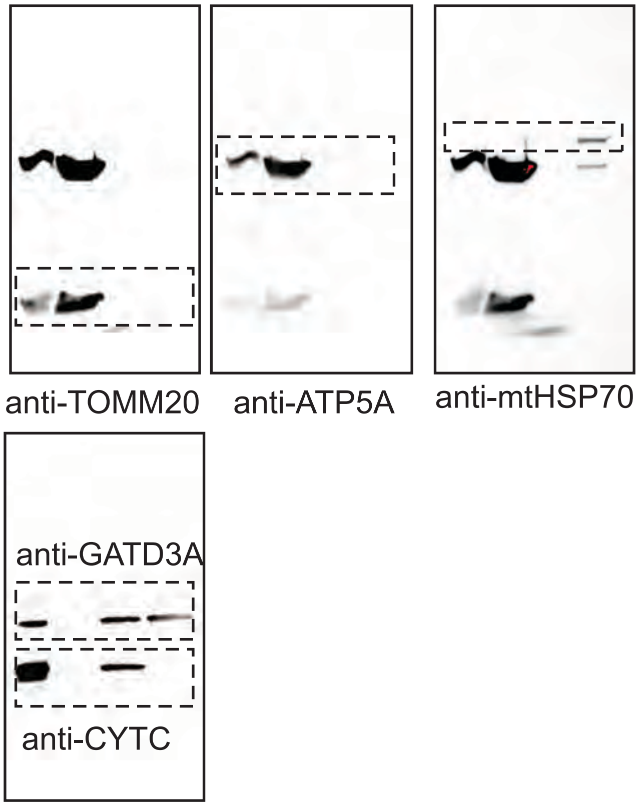

Fig 3D

DNA, GO, Full Blot

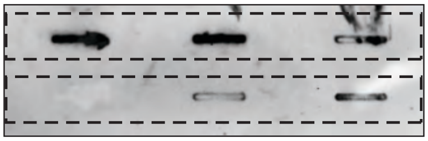

Protein, GO, Full Blot

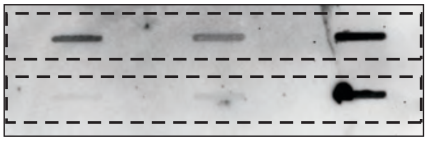

DNA, MGO, Full Blot

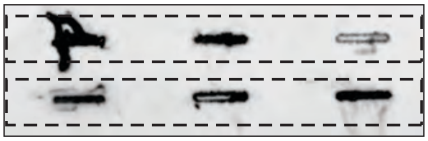

Protein, MGO, Full Blot

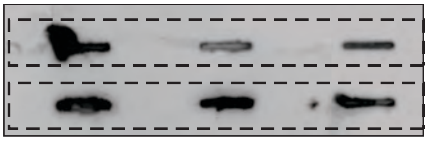

Fig 3E and Fig S4A

2 month old mice

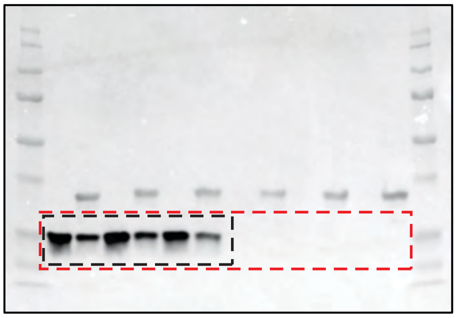

anti-GATD3A

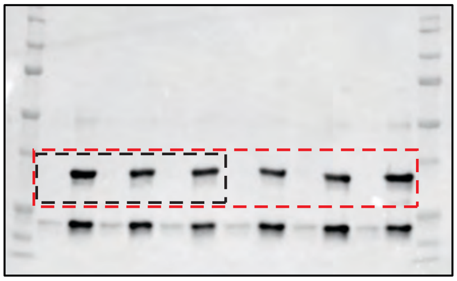

anti-DJ1

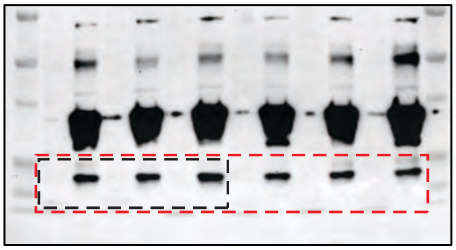

anti-GLO1

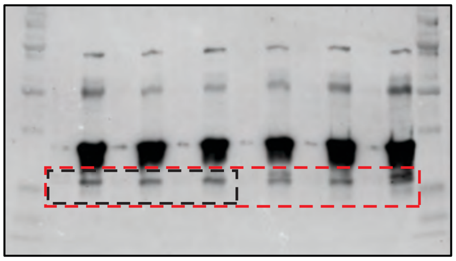

anti-GLO2

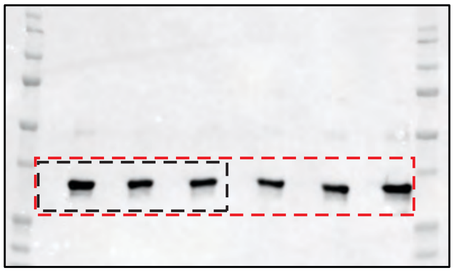

anti-GAPDH

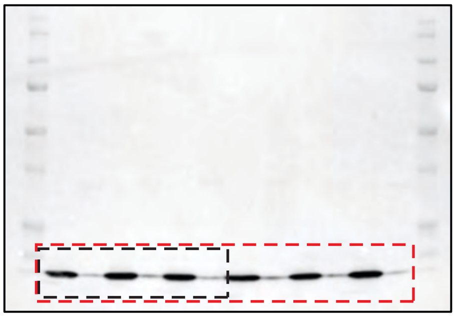

anti-COXIV

23 month old mice

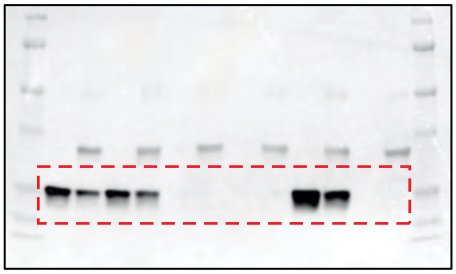

anti-GATD3A

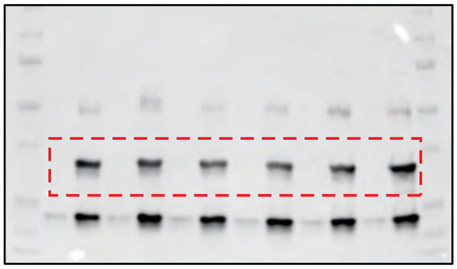

anti-DJ1

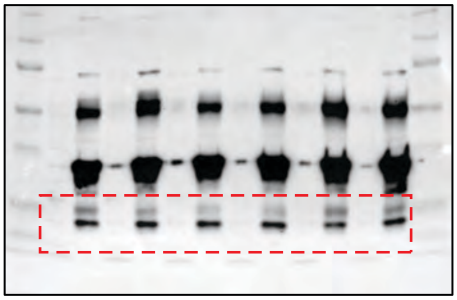

anti-GLO1

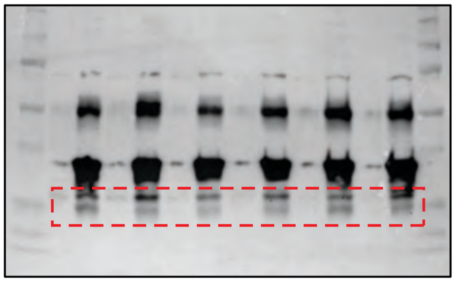

anti-GLO2

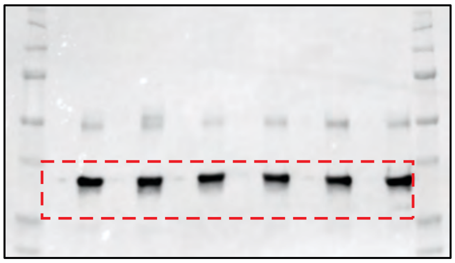

anti-GAPDH

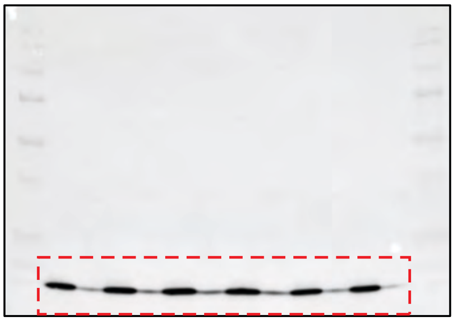

anti-COXIV

Fig 4A

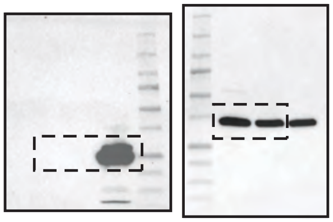

anti-FLAG anti-GAPDH

Fig 4B

Gatd3a (wildtype) Gatd3a (FLAG/FLAG)

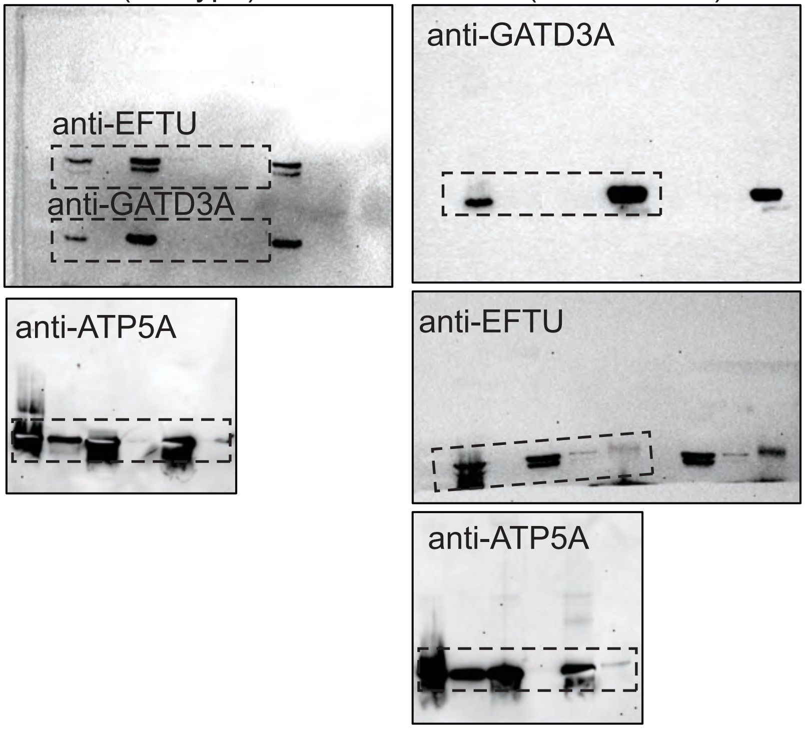

Fig 4G

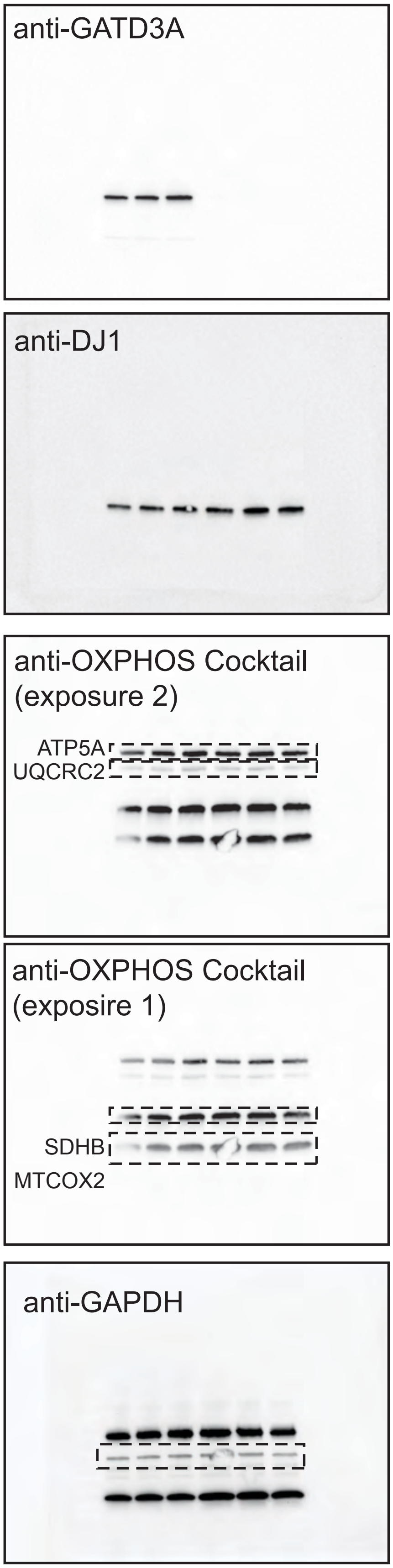

Fig S1D

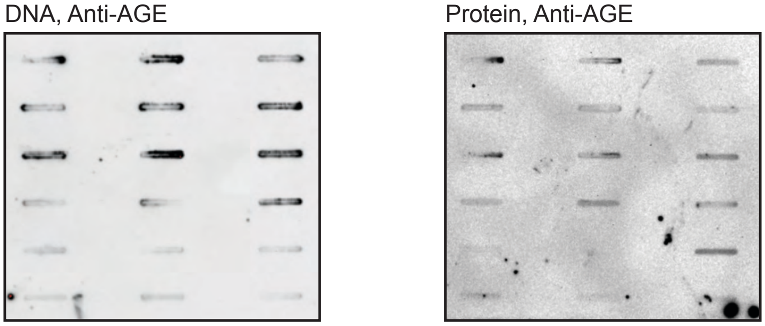

Supplement: Supplementary file 2 — Additional file 2. Uncropped gel images with indicated figures. [file 12915_2022_1267_MOESM2_ESM.pdf]
